# Supplementary material for: The Suitability of RNA from Positive SARS-CoV-2 Rapid Antigen Tests for Whole Virus Genome Sequencing and Variant Identification to Maintain Genomic Surveillance
Source: Diagnostics (Basel). 2023 Dec 7;13(24):3618. doi: 10.3390/diagnostics13243618 (PMC10742923; doi:10.3390/diagnostics13243618)
Supplement: Supplementary file 1 [file diagnostics-13-03618-s001.zip › diagnostics-2665763-supplementary.pdf]

Supplementary Table S1

Month	Sample	Valore CT	Lineage	N_consensus	QC_status	Variante
June	3422036442	28,69	Unassigned	31,9	Failed	Omicron
June	3422036565	28,54	BA.2.9	23,9	Failed	Omicron2
June	3422037224	28,46	BE.1	16	Failed	Omicron5
June	3422036803	28,29	BA.2	22,3	Failed	Omicron2
June	3422036568	28,14	BA.4	27,8	Failed	Omicron2
June	3422036562	28,11	BA.4.1	24,6	Failed	Omicron4
June	3422036560	28,04	BA.2	22,8	Failed	Omicron2
June	3422037209	27,93	BA.5.1	12,1	Failed	Omicron5
June	3422036610	27,9	Unassigned	34,2	Failed	Omicron
June	3422036598	27,84	Unassigned	31,9	Failed	Omicron
June	3422037230	27,63	BA.2	13,7	Failed	Omicron2
June	3422037225	27,62	BE.1	8,7	Failed	Omicron5
June	3422037217	27,55	BA.5.1	9,5	Failed	Omicron5
June	3422036877	27,43	BA.2	19,5	Failed	Omicron2
June	3422037222	27,43	Unassigned	34,5	Failed	Omicron
June	3422036809	27,39	BA.4	29,4	Failed	Omicron2
June	3422036445	27,29	BA.5.1	11,9	Failed	Omicron5
June	3422036443	27,08	BA.5	19,9	Failed	Omicron2
June	3422037199	27,07	BA.2.12.1	20,2	Failed	Omicron2
June	3422036383	27,01	BA.2	11,8	Failed	Omicron2
June	3422036573	26,99	BA.2.9	23,9	Failed	Omicron2
June	3422036379	26,89	BA.5.1.35	14,7	Failed	Omicron5
June	3422037226	26,86	BA.2.3.15	12,1	Failed	Omicron2
June	3422036588	26,82	BA.2	22,9	Failed	Omicron2
June	3422037211	26,8	BA.2.70	6,3	Failed	Omicron2
June	3422037234	26,79	BA.2.9	7,1	Failed	Omicron2
June	3422036566	26,78	BA.2	21,3	Failed	Omicron2
June	3422036604	26,7	BA.2	21,6	Failed	Omicron2
June	3422036611	26,68	BA.2	24,1	Failed	Omicron2
June	3422036605	26,63	BA.5	18	Failed	Omicron5
June	3422036417	26,53	BF.1	12,5	Failed	Omicron5
June	3422036884	26,5	BA.2	7,1	Failed	Omicron2
June	3422036613	26,35	BE.1	19,8	Failed	Omicron5
June	3422037220	26,28	BA.2	10	Failed	Omicron2
June	3422036579	26,17	BA.2	22,8	Failed	Omicron2
June	3422036585	25,97	BA.2	18,5	Failed	Omicron2
June	3422036576	25,82	BA.2.10	18,7	Failed	Omicron2
June	3422036788	25,73	BA.2.10	4,8	Passed	Omicron2
June	3422036813	25,71	BA.2	16,2	Failed	Omicron2
June	3422037241	25,67	BA.2	6,2	Failed	Omicron2
June	3422036583	25,3	BA.2	13,9	Failed	Omicron2
June	3422036609	25,27	BA.5.1	15,9	Failed	Omicron5
June	3422036577	25,17	BA.2	15,4	Failed	Omicron2
June	3422036602	25,14	BA.2.3.15	14,2	Failed	Omicron2
June	3422036896	25,1	BA.5.1	9,1	Failed	Omicron5
June	3422036785	25	BA.2	10,2	Failed	Omicron2
June	3422037213	24,88	BA.5.1	7,4	Failed	Omicron5
June	3422036806	24,75	BA.5.1	3,8	Passed	Omicron5
June	3422037205	24,74	BA.2.13	4,3	Passed	Omicron2
June	3422036388	24,64	BA.5.1	7,5	Failed	Omicron5
June	3422036596	24,56	BA.2	12,9	Failed	Omicron2
June	3422036584	24,36	BA.2	13,6	Failed	Omicron2
June	3422036370	24,21	BA.4	6,7	Failed	Omicron4
June	3422036903	24,15	BA.5.1	2,8	Passed	Omicron5

June	3422036789	24,1	BE.1	2,7	Passed	Omicron5
June	3422036589	24,04	BA.2	7,3	Failed	Omicron2
June	3422036580	23,93	BA.2	7,7	Failed	Omicron2
June	3422036414	23,86	BA.4	19	Failed	Omicron4
June	3422036882	23,86	BA.2.12.1	2,9	Passed	Omicron2
June	3422037235	23,78	BA.4	3,2	Passed	Omicron4
June	3422036452	23,76	BA.5.1.1	5,6	Failed	Omicron5
June	3422038513	23,7	BA.4.1	6,3	Failed	Omicron4
June	3422036590	23,67	BA.2.52	7,4	Failed	Omicron2
June	3422036430	23,61	BA.2	5,8	Failed	Omicron2
June	3422036380	23,58	BA.5.1	8,7	Failed	Omicron5
June	3422036375	23,56	BA.2.52	2,7	Passed	Omicron2
June	3422036886	23,53	BE.1	2,9	Passed	Omicron5
June	3422037215	23,45	BA.2	1,9	Passed	Omicron2
June	3422036403	23,41	BA.5.1	9,4	Failed	Omicron5
June	3422036582	23,32	BA.5.1	8,2	Failed	Omicron5
June	3422036887	23,32	BA.5.1	6,6	Failed	Omicron5
June	3422036593	23,31	BA.5.1	8,2	Failed	Omicron5
June	3422036898	23,09	BA.5.1	4,1	Passed	Omicron5
June	3422037202	23,02	BA.2	1,2	Passed	Omicron2
June	3422037221	22,99	BA.2	1,9	Passed	Omicron2
June	3422038523	22,98	BA.5.1	5,5	Failed	Omicron5
June	3422036406	22,81	BA.2	3,7	Passed	Omicron2
June	3422037204	22,77	BA.2	2,5	Passed	Omicron2
June	3422037207	22,77	BA.2	4,7	Passed	Omicron2
June	3422037239	22,62	BA.5.1	1,5	Passed	Omicron5
June	3422037210	22,55	BA.5.1	1,3	Passed	Omicron5
June	3422036446	22,48	BA.5.1	6,2	Failed	Omicron5
June	3422036895	22,48	BA.4	0,8	Passed	Omicron4
June	3422036569	22,43	BA.5.1.1	5,3	Failed	Omicron5
June	3422036434	22,35	BA.5.1.24	3,5	Passed	Omicron5
June	3422037203	22,04	BA.2.3.15	0,6	Passed	Omicron2
June	3422037232	21,9	BA.2	1,6	Passed	Omicron2
June	3422036571	21,81	BA.4	4,7	Passed	Omicron4
June	3422036372	21,79	BA.2.9	4	Passed	Omicron2
June	3422036592	21,75	BA.2.3.15	5,1	Failed	Omicron2
June	3422037216	21,75	BA.5.1	1,2	Passed	Omicron5
June	3422036811	21,71	BA.2.9	1,3	Passed	Omicron2
June	3422036597	21,68	BA.2.54	4,8	Passed	Omicron2
June	3422037260	21,65	BA.2.13	1,3	Passed	Omicron2
June	3422037227	21,54	BA.2	6,4	Failed	Omicron2
June	3422036448	21,37	BA.2.70	3,2	Passed	Omicron2
June	3422036401	21,36	BA.5.1.2	0,6	Passed	Omicron5
June	3422036790	21,34	BA.2.23	4,9	Passed	Omicron2
June	3422036816	21,28	BA.2.9	0,9	Passed	Omicron2
June	3422036570	21,25	BA.2	3,3	Passed	Omicron2
June	3422036595	21,22	BA.5.1	3,8	Passed	Omicron5
June	3422036587	21,2	BA.2.3	3,4	Passed	Omicron2
June	3422036586	21,13	BA.5.1	3,4	Passed	Omicron5
June	3422036869	21,06	BA.2	1,4	Passed	Omicron2
June	3422037219	21,06	BA.5.1.3	2,2	Passed	Omicron5
June	3422037236	20,97	BA.2	0,8	Passed	Omicron2
June	3422036804	20,9	BA.5.1	1,8	Passed	Omicron5
June	3422038502	20,87	BA.2	25,6	Failed	Omicron2
June	3422036897	20,84	BA.5.1	1,5	Passed	Omicron5
June	3422036428	20,82	BA.2.44	2,2	Passed	Omicron2

June	3422037246	20,75	BA.2	1	Passed	Omicron2
June	3422036601	20,73	BA.2.9	3,8	Passed	Omicron2
June	3422036394	20,72	BA.5.1	1	Passed	Omicron5
June	3422036815	20,69	BA.2	0,2	Passed	Omicron2
June	3422037266	20,67	BA.2.56	1,1	Passed	Omicron2
June	3422036399	20,55	BA.2	1,8	Passed	Omicron2
June	3422036800	20,54	BA.5.1	0,9	Passed	Omicron5
June	3422036412	20,48	BA.5.1	0,7	Passed	Omicron5
June	3422037250	20,46	BA.2.3.15	0,9	Passed	Omicron2
June	3422036603	20,45	BA.2	1,5	Passed	Omicron2
June	3422036395	20,34	BA.5.1	0,3	Passed	Omicron5
June	3422037228	20,33	BA.2	0,9	Passed	Omicron2
June	3422036572	20,31	BA.2	0,6	Passed	Omicron2
June	3422036382	20,1	BA.2	1	Passed	Omicron2
June	3422036814	20,1	BA.2.10	0,5	Passed	Omicron2
June	3422036866	20,08	BA.2	0,8	Passed	Omicron2
June	3422036575	20,05	BA.2	1,7	Passed	Omicron2
June	3422036439	19,84	BA.5.1	1,8	Passed	Omicron5
June	3422036904	19,82	BA.5.1	0,3	Passed	Omicron5
June	3422036784	19,81	BA.2	0,2	Passed	Omicron2
June	3422036787	19,68	BA.2.9	1	Passed	Omicron2
June	3422036893	19,67	BA.2.9	0,3	Passed	Omicron2
June	3422036880	19,65	BA.5.1.2	0,8	Passed	Omicron5
June	3422036441	19,61	BA.2	1,1	Passed	Omicron2
June	3422038512	19,59	BA.2	0,8	Passed	Omicron2
June	3422036390	19,57	BA.2	0,7	Passed	Omicron2
June	3422036429	19,56	BA.2.10	0,6	Passed	Omicron2
June	3422037248	19,39	BA.2	0,8	Passed	Omicron2
June	3422036864	19,31	BA.4	0	Passed	Omicron4
June	3422036599	19,3	BA.2	2	Passed	Omicron2
June	3422036792	19,28	BA.2	0,4	Passed	Omicron2
June	3422036883	19,24	BA.2	0,2	Passed	Omicron2
June	3422036386	19,14	BA.2	0,4	Passed	Omicron2
June	3422036591	19,12	BA.5.1	0,6	Passed	Omicron5
June	3422036377	18,94	BA.2.9	0,4	Passed	Omicron2
June	3422036798	18,83	BA.2.9	0,4	Passed	Omicron2
June	3422036890	18,69	BA.5.1.1	0,2	Passed	Omicron5
June	3422036812	18,56	BA.2.10	0,4	Passed	Omicron2
June	3422036449	18,39	BA.5.2.1	0,4	Passed	Omicron5
June	3422036799	18,27	BA.2	0,4	Passed	Omicron2
June	3422036402	18,24	BA.5.1.2	0,2	Passed	Omicron5
June	3422037003	18,15	BA.2	0,1	Passed	Omicron2
June	3422036879	18,14	BA.5.1.2	1,3	Passed	Omicron5
June	3422036416	18,08	BA.5.1	0,2	Passed	Omicron5
June	3422036410	18,06	BA.5.1.10	0,2	Passed	Omicron5
June	3422036796	17,93	BA.2	0,2	Passed	Omicron2
June	3422037233	17,92	BA.2.71	0,2	Passed	Omicron2
June	3422036899	17,91	BA.2	0,2	Passed	Omicron2
June	3422037286	17,79	BA.5.1	0,4	Passed	Omicron5
June	3422036435	17,69	BA.2.52	0,4	Passed	Omicron2
June	3422036578	17,66	BA.2	2,7	Passed	Omicron2
June	3422036404	17,59	BA.5.1.2	0,1	Passed	Omicron5
June	3422037212	17,49	BA.5.1	0,2	Passed	Omicron5
June	3422036378	17,48	BA.2.9	0,4	Passed	Omicron2
June	3422036817	17,48	BA.2	0,4	Passed	Omicron2
June	3422036865	17,48	BA.2	0,1	Passed	Omicron2

June	3422036810	17,44	BA.5.1	0,2	Passed	Omicron5
June	3422036805	17,42	BA.5.1	1,2	Passed	Omicron5
June	3422036433	17,41	BF.27	0,3	Passed	Omicron5
June	3422036797	17,4	BA.2	0,4	Passed	Omicron2
June	3422036436	17,32	BA.2.23	0,4	Passed	Omicron2
June	3422036561	17,3	BA.4.1	1,1	Passed	Omicron4
June	3422036889	17,16	BA.5.1	0,2	Passed	Omicron5
June	3422036374	17,13	BA.2.23	0,3	Passed	Omicron2
June	3422036381	16,97	BA.5.1	0,6	Passed	Omicron5
June	3422037287	16,92	BA.5.1	0,2	Passed	Omicron5
June	3422036907	16,89	BA.4.1	0	Passed	Omicron4
June	3422036369	16,8	BA.5.1	0,2	Passed	Omicron5
June	3422036801	16,77	BA.4	1,3	Passed	Omicron4
June	3422036901	16,76	BA.2	0,4	Passed	Omicron2
June	3422036393	16,7	BA.5.1	0,2	Passed	Omicron5
June	3422036875	16,66	BA.5.1	0,3	Passed	Omicron5
June	3422036400	16,61	BA.2	0,4	Passed	Omicron2
June	3422036905	16,59	BA.2	0,3	Passed	Omicron2
June	3422036881	16,44	BA.2.68	0,4	Passed	Omicron2
June	3422037223	16,43	BA.2	0,4	Passed	Omicron2
June	3422036793	16,39	BA.2.3.15	0,1	Passed	Omicron2
June	3422036807	16,31	BA.5.1	0,7	Passed	Omicron5
June	3422036385	16,29	BA.2	0,4	Passed	Omicron2
June	3422036415	16,08	BA.5.1	0,4	Passed	Omicron5
June	3422036786	16,05	BA.2.36	0,4	Passed	Omicron2
June	3422037206	16,04	BA.2.9	0,4	Passed	Omicron2
June	3422038518	15,95	BA.5.1	0,2	Passed	Omicron5
June	3422036891	15,82	BA.5.1.1	0,3	Passed	Omicron5
June	3422036902	15,64	BA.4.1	0,4	Passed	Omicron4
June	3422036450	15,55	BA.5.2.1	0,3	Passed	Omicron5
June	3422036451	15,55	BA.5.1	0,4	Passed	Omicron5
June	3422036791	15,53	BA.4	0,2	Passed	Omicron4
June	3422037002	15,46	BA.2	0,4	Passed	Omicron2
June	3422036447	15,28	BA.5.1	0,4	Passed	Omicron5
June	3422036409	15,26	BA.2.10	0,4	Passed	Omicron2
June	3422036795	15,24	BA.5.1	0,2	Passed	Omicron5
June	3422036870	15,23	BA.5.1	0,2	Passed	Omicron5
June	3422036868	15,2	BA.5.1	0,4	Passed	Omicron5
June	3422036808	15,08	BA.5.1.1	0,1	Passed	Omicron5
June	3422036862	14,91	BA.2	0,1	Passed	Omicron2
June	3422036873	14,88	BA.5.1	0,4	Passed	Omicron5
June	3422036437	14,55	BA.2	0,2	Passed	Omicron2
June	3422036872	14,29	BA.5.1	0,3	Passed	Omicron5
June	3422038484	14,19	BA.5.1.23	0	Passed	Omicron5
June	3422036794	14,18	BA.2.3.15	0,1	Passed	Omicron2
June	3422036373	13,98	BA.2.9	0,3	Passed	Omicron2
June	3422036391	13,95	BA.2.52	0,4	Passed	Omicron2
June	3422036863	13,83	BA.4	0,2	Passed	Omicron4
June	3422036387	13,66	BA.5.1	0,2	Passed	Omicron5
June	3422036876	13,62	BA.5.1	0,2	Passed	Omicron5
June	3422036384	13,41	BA.2	0,2	Passed	Omicron2
June	3422036431	13,2	BA.5.1	0,2	Passed	Omicron5
June	3422036408	13,19	BA.2	0,4	Passed	Omicron2
June	3422036371	13,15	BA.5.2	0,4	Passed	Omicron5
June	3422036885	13,11	BA.2	0,2	Passed	Omicron2
June	3422036411	13,06	BA.2.3.14	2,9	Passed	Omicron2

June	3422036874	12,88	BA.2.3.15	0,4	Passed	Omicron2
June	3422036888	12,66	BE.1	0,1	Passed	Omicron5
June	3422036432	12,6	BA.5.1	0,4	Passed	Omicron5
June	3422036444	12,59	BA.2.70	0,2	Passed	Omicron2
June	3422036894	12,03	BA.5.1.1	0,2	Passed	Omicron5
June	3422036389	11,95	BA.2	29,3	Failed	Omicron2
June	3422036802	11,76	BA.2	0,3	Passed	Omicron2
June	3422036392	11,64	BA.2.52	0,2	Passed	Omicron2
June	3422036581	11,38	BA.5.1	0,4	Passed	Omicron5
June	3422036376	10,84	BA.2	0,2	Passed	Omicron2

Supplementary Table S2

Month	Sample	Valore CT	Lineage	N_consensus	QC_status	Variante
July	22053762	31,58	BA.2	1918 (6.4%)	Failed	Omicron2
July	22051634	30,01	BA.5	7048 (23.9%)	Failed	Omicron5
July	22051313	29,6	BA.2	4055 (13.6%)	Failed	Omicron2
July	22052871	29,42	Unassigned	10654 (36.1%)	Failed	Omicron
July	22051309	28,36	BA.5.2.1	3398 (11.4%)	Failed	Omicron5
July	22053625	28,19	BA.2	1616 (5.4%)	Failed	Omicron2
July	22047197	28,12	Unassigned	9065 (30.7%)	Failed	Omicron
July	22049749	30,75	BA.5.2	118 (0.4%)	Passed	Omicron5
July	22053386	29,9	BA.2	1483 (5.0%)	Passed	Omicron2
July	22054499	29,69	BA.5.1	735 (2.5%)	Passed	Omicron5
July	22052695	28,95	BA.5.1	1457 (4.9%)	Passed	Omicron5
July	22053622	28,26	BA.5	1056 (3.5%)	Passed	Omicron5
July	22051635	27,91	BA.4	5776 (19.4%)	Failed	Omicron4
July	22046637	27,74	BA.5.2	77 (0.3%)	Passed	Omicron5
July	22050694	27,73	BA.4	2945 (9.9%)	Failed	Omicron4
July	22050682	27,59	BF.4	2520 (8.4%)	Failed	Omicron5
July	22052867	26,9	BA.5.2.1	846 (2.8%)	Passed	Omicron5
July	22052719	26,45	BA.5.2	3227 (10.8%)	Failed	Omicron5
July	22052877	26,32	BA.5	714 (2.4%)	Passed	Omicron5
July	22051306	25,56	BA.5.1.10	2402 (8.1%)	Failed	Omicron5
July	22047897	25,51	BA.5.2.1	3024 (10.1%)	Failed	Omicron5
July	22054287	25,38	BA.5.1	1048 (3.5%)	Passed	Omicron5
July	22052872	25,27	BF.1	2777 (9.3%)	Failed	Omicron5
July	22047899	25,11	BA.5.1	2591 (8.8%)	Failed	Omicron5
July	22053764	25,1	BE.1	516 (1.7%)	Passed	Omicron5
July	22052704	25,08	BA.5.1.24	3182 (10.7%)	Failed	Omicron5
July	22051636	24,87	BA.5.1	2073 (7.0%)	Failed	Omicron5
July	22053294	24,77	BA.5.1	481 (1.6%)	Passed	Omicron5
July	22053380	24,55	BA.5.1	344 (1.2%)	Passed	Omicron5
July	22047385	24,41	BA.5.1	3001 (10.0%)	Failed	Omicron5
July	22052213	24,37	BA.5.1	99 (0.3%)	Passed	Omicron5
July	22053637	23,8	BA.5.1	287 (1.0%)	Passed	Omicron5
July	22053385	23,64	BE.1	390 (1.3%)	Passed	Omicron5
July	22054282	23,56	BA.5.1	315 (1.1%)	Passed	Omicron5
July	22053291	23,49	BA.5.1.23	298 (1.0%)	Passed	Omicron5
July	22050714	23,46	BA.5.1	1486 (5.0%)	Passed	Omicron5
July	22053623	23,38	BA.5.1	568 (1.9%)	Passed	Omicron5
July	22047907	23,29	BA.2.9.3	1889 (6.3%)	Failed	Omicron2
July	22052698	23,28	BA.5.1	114 (0.4%)	Passed	Omicron5
July	22051310	22,96	BA.5.1	713 (2.4%)	Passed	Omicron5
July	22052874	22,92	BA.5.1	472 (1.6%)	Passed	Omicron5
July	22052875	22,88	BA.5.1	1651 (5.6%)	Failed	Omicron5
July	22052216	22,73	BE.1.1	877 (2.9%)	Passed	Omicron5
July	22051642	22,72	BE.1	1539 (5.2%)	Failed	Omicron5
July	22054212	22,52	BE.1	243 (0.8%)	Passed	Omicron5
July	22052697	22,48	BA.5.2	1025 (3.4%)	Passed	Omicron5
July	22052699	22,09	BA.5.1	1157 (3.9%)	Passed	Omicron5
July	22053279	21,92	BA.5.1	222 (0.7%)	Passed	Omicron5
July	22049747	21,69	BA.5.1	133 (0.4%)	Passed	Omicron5
July	22053295	21,59	BA.5.2.1	227 (0.8%)	Passed	Omicron5
July	22046635	21,46	BA.5.1	76 (0.3%)	Passed	Omicron5
July	22049788	21,46	BE.1.1	580 (1.9%)	Passed	Omicron5
July	22053293	21,46	BA.5.1	350 (1.2%)	Passed	Omicron5
July	22053283	21,41	BA.5.1.2	450 (1.5%)	Passed	Omicron5
July	22050715	21,28	BA.5.1	181 (0.6%)	Passed	Omicron5
July	22051312	21,24	BA.5.2	1076 (3.6%)	Passed	Omicron5
July	22051632	21,2	BA.5.2	333 (1.1%)	Passed	Omicron5
July	22047367	21,14	BA.5.1	1224 (4.1%)	Passed	Omicron5
July	22053382	21,13	BA.5.2.1	380 (1.3%)	Passed	Omicron5
July	22054281	21,07	BA.5.1	205 (0.7%)	Passed	Omicron5
July	22047366	20,93	BA.5.1	657 (2.2%)	Passed	Omicron5
July	22053778	20,81	BA.5.1.10	744 (2.5%)	Passed	Omicron5
July	22047375	20,8	BA.2.1	887 (3.0%)	Passed	Omicron2
July	22050697	20,79	BA.5.2	107 (0.4%)	Passed	Omicron5
July	22052706	20,65	BA.5.1.24	331 (1.1%)	Passed	Omicron5
July	22049790	20,63	BA.5.1	120 (0.4%)	Passed	Omicron5
July	22047198	20,49	BA.5.1.10	201 (0.7%)	Passed	Omicron5
July	22052878	20,15	BA.5.1	116 (0.4%)	Passed	Omicron5
July	22053648	20,08	BF.7	286 (1.0%)	Passed	Omicron5
July	22053274	19,68	BA.5.1	538 (1.8%)	Passed	Omicron5
July	22051305	19,63	BA.5.1.10	114 (0.4%)	Passed	Omicron5
July	22053394	19,51	BE.1.3	186 (0.6%)	Passed	Omicron5
July	22053620	19,51	BA.5.1	264 (0.9%)	Passed	Omicron5

July	22054284	19,51	BA.5.2.59	154 (0.5%)	Passed	Omicron5
July	22052696	19,5	BA.5.1	273 (0.9%)	Passed	Omicron5
July	22052870	19,41	BA.5.1	460 (1.5%)	Passed	Omicron5
July	22053624	19,39	BA.5.1	285 (1.0%)	Passed	Omicron5
July	22050688	19,36	BA.5.1.30	118 (0.4%)	Passed	Omicron5
July	22052880	19,19	BA.5.2.1	167 (0.6%)	Passed	Omicron5
July	22049647	19,02	BA.5.1	119 (0.4%)	Passed	Omicron5
July	22053785	18,91	BA.5.1.23	336 (1.1%)	Passed	Omicron5
July	22053392	18,85	BA.5.1.10	174 (0.6%)	Passed	Omicron5
July	22046891	18,79	BA.4	87 (0.3%)	Passed	Omicron4
July	22049795	18,76	BA.5.1	313 (1.1%)	Passed	Omicron5
July	22047193	18,69	BA.5.1.30	117 (0.4%)	Passed	Omicron5
July	22053631	18,65	BA.5.1	193 (0.6%)	Passed	Omicron5
July	22047892	18,56	BA.5.1	199 (0.7%)	Passed	Omicron5
July	22054283	18,51	BA.5.1	430 (1.4%)	Passed	Omicron5
July	22047895	18,5	BA.5.1	11 (0.0%)	Passed	Omicron5
July	22046889	18,4	BA.5.2	1665 (5.6%)	Failed	Omicron5
July	22053388	18,32	BE.1	349 (1.2%)	Passed	Omicron5
July	22052215	18,1	BA.5.1.10	280 (0.9%)	Passed	Omicron5
July	22047365	18,03	BA.5.1	77 (0.3%)	Passed	Omicron5
July	22049793	18,02	BA.5.2.1	287 (1.0%)	Passed	Omicron5
July	22054286	17,83	BA.5.2	117 (0.4%)	Passed	Omicron5
July	22051304	17,68	BA.5.1.10	62 (0.2%)	Passed	Omicron5
July	22053638	17,67	BA.5.1	29 (0.1%)	Passed	Omicron5
July	22053766	17,62	BA.5.2.9	58 (0.2%)	Passed	Omicron5
July	22047362	17,49	BA.5.1	218 (0.7%)	Passed	Omicron5
July	22049746	17,47	BA.4.6	95 (0.3%)	Passed	Omicron4
July	22054502	17,19	BA.5.1	113 (0.4%)	Passed	Omicron5
July	22054501	17,09	BA.5.1.3	99 (0.3%)	Passed	Omicron5
July	22054513	17,06	BA.4.7	335 (1.1%)	Passed	Omicron4
July	22049659	16,83	BA.5.2.1	118 (0.4%)	Passed	Omicron5
July	22052881	16,82	BA.5.1	120 (0.4%)	Passed	Omicron5
July	22053864	16,8	BA.5.2.1	116 (0.4%)	Passed	Omicron5
July	22054288	16,75	BA.5.1	73 (0.2%)	Passed	Omicron5
July	22053269	16,72	BA.5.2	117 (0.4%)	Passed	Omicron5
July	22054500	16,71	BA.5.1.3	28 (0.1%)	Passed	Omicron5
July	22046634	16,51	BA.5.2.12	5907 (19.8%)	Failed	Omicron5
July	22046886	16,49	BA.5.1.10	114 (0.4%)	Passed	Omicron5
July	22053769	16,47	BA.5.2	74 (0.2%)	Passed	Omicron5
July	22046887	16,41	BA.5.1.1	78 (0.3%)	Passed	Omicron5
July	22047909	16,4	BA.5.1	65 (0.2%)	Passed	Omicron5
July	22052707	16,4	BA.5.1	82 (0.3%)	Passed	Omicron5
July	22050685	16,32	BA.5.1	11 (0.0%)	Passed	Omicron5
July	22049750	16,25	BA.5.1	921 (3.1%)	Passed	Omicron5
July	22054498	16,25	XAZ	68 (0.2%)	Passed	Ricombinante
July	22050683	16,2	BA.5.1	99 (0.3%)	Passed	Omicron5
July	22051311	16,19	BA.5.1	63 (0.2%)	Passed	Omicron5
July	22047360	15,82	BA.5.1	69 (0.2%)	Passed	Omicron5
July	22049748	15,77	BA.5.1	6507 (21.8%)	Failed	Omicron5
July	22053640	15,72	BA.5.1	116 (0.4%)	Passed	Omicron5
July	22047361	15,47	BA.5.1	67 (0.2%)	Passed	Omicron5
July	22053278	15,47	BF.2	69 (0.2%)	Passed	Omicron5
July	22054511	15,38	BA.4	69 (0.2%)	Passed	Omicron4
July	22052700	15,37	BA.5.1	77 (0.3%)	Passed	Omicron5
July	22047371	15,19	BA.5.1	114 (0.4%)	Passed	Omicron5
July	22046877	15,06	BA.5.2	81 (0.3%)	Passed	Omicron5
July	22051648	15,06	BE.1	73 (0.2%)	Passed	Omicron5
July	22049791	15,01	BA.5.1.10	889 (3.0%)	Passed	Omicron5
July	22053387	14,96	BA.5.2.1	68 (0.2%)	Passed	Omicron5
July	22047201	14,87	BA.5.1	88 (0.3%)	Passed	Omicron5
July	22054503	14,81	BA.5.1	73 (0.2%)	Passed	Omicron5
July	22047199	14,71	BA.5.2	118 (0.4%)	Passed	Omicron5
July	22054289	14,68	BA.5.2.44	16 (0.1%)	Passed	Omicron5
July	22051307	14,49	BA.5.1	67 (0.2%)	Passed	Omicron5
July	22047363	14,48	BA.5.1	117 (0.4%)	Passed	Omicron5
July	22051640	14,42	BA.5.1	862 (2.9%)	Passed	Omicron5
July	22052218	14,34	BA.5.1	69 (0.2%)	Passed	Omicron5
July	22054510	14,18	BA.4	118 (0.4%)	Passed	Omicron4
July	22053381	14,16	BA.5.1.30	116 (0.4%)	Passed	Omicron5
July	22053384	14,01	BA.2.52	74 (0.2%)	Passed	Omicron2
July	22047917	13,75	BA.5.1	491 (1.6%)	Passed	Omicron5
July	22052221	13,57	BA.5.2	98 (0.3%)	Passed	Omicron5
July	22052217	13,52	BA.5.2	114 (0.4%)	Passed	Omicron5
July	22052214	13,37	BA.5.1.15	3807 (12.8%)	Failed	Omicron5
July	22052219	13,1	BA.5.1	76 (0.3%)	Passed	Omicron5

July	22051308	13,05	BA.5.1.30	118 (0.4%)	Passed	Omicron5
July	22051630	12,93	BA.5.2	71 (0.2%)	Passed	Omicron5
July	22051629	12,89	BE.1	778 (2.6%)	Passed	Omicron5
July	22046890	12,74	BE.1	81 (0.3%)	Passed	Omicron5
July	22047195	12,49	BA.5.1	70 (0.2%)	Passed	Omicron5
July	22049654	12,32	BE.1	76 (0.3%)	Passed	Omicron5
July	22052220	11,97	BA.5.1.10	69 (0.2%)	Passed	Omicron5
July	22052212	11,9	BE.2	87 (0.3%)	Passed	Omicron5
July	22053765	11,9	BA.5.1	458 (1.5%)	Passed	Omicron5
July	22050716	11,67	BA.5.1	42 (0.1%)	Passed	Omicron5
July	22054211	11,49	BE.3	15 (0.1%)	Passed	Omicron5

Supplementary Table S3

Month	Sample	Valore CT	Lineage	N_consensus	QC_status	Variante
August	22055422	35,95	BA.5.1	293 (1.0%)	Passed	Omicron5
August	22055399	30,61	BA.5.1	1095 (3.7%)	Passed	Omicron5
August	22055379	30,55	BA.5.1	362 (1.2%)	Passed	Omicron5
August	22055406	30,53	BA.5.1	1081 (3.6%)	Passed	Omicron5
August	22055410	30,5	BA.5.1	222 (0.7%)	Passed	Omicron5
August	22057641	30,49	BA.5.1	2928 (9.8%)	Failed	Omicron5
August	22055397	29,99	BA.5.1.3	1020 (3.4%)	Passed	Omicron5
August	22055852	29,59	BA.4.1	2116 (7.1%)	Failed	Omicron4
August	22055371	29,27	BA.5.2.1	883 (3.0%)	Passed	Omicron5
August	22055363	29,23	BA.5.1	241 (0.8%)	Passed	Omicron5
August	22055861	28,94	BA.5.1	2366 (7.9%)	Failed	Omicron5
August	22057978	28,83	BA.5.2	998 (3.4%)	Passed	Omicron5
August	22057043	28,56	BA.5.2	341 (1.1%)	Passed	Omicron5
August	22057122	28,04	BA.5.1.10	1659 (5.6%)	Failed	Omicron5
August	22055409	27,94	BA.5.1	24 (0.1%)	Passed	Omicron5
August	22055854	27,56	Unassigned	13261 (44.5%)	Failed	Omicron
August	22055362	27,54	BA.5.1	408 (1.4%)	Passed	Omicron5
August	22058426	27,54	BA.5.2	2121 (7.1%)	Failed	Omicron5
August	22056498	27,32	BA.5.1.22	1170 (3.9%)	Passed	Omicron5
August	22056491	27,3	BA.5.2	1261 (4.2%)	Passed	Omicron5
August	22055417	27,28	BA.5.1	731 (2.5%)	Passed	Omicron5
August	22058324	27,24	BA.5.1	277 (0.9%)	Passed	Omicron5
August	22057113	27,17	BA.5.1	1690 (5.7%)	Failed	Omicron5
August	22055420	27,07	BA.5.1	1012 (3.4%)	Passed	Omicron5
August	22056222	27,04	BA.5.2	672 (2.3%)	Passed	Omicron5
August	22056494	27,01	BA.5.2.22	1148 (3.9%)	Passed	Omicron5
August	22055337	26,99	BA.5.1	1018 (3.4%)	Passed	Omicron5
August	22055859	26,85	BA.5.2	1403 (4.7%)	Passed	Omicron5
August	22055407	26,8	BA.5.1	552 (1.9%)	Passed	Omicron5
August	22055390	26,69	BA.5.1	833 (2.8%)	Passed	Omicron5
August	22055941	26,69	BA.5.1	618 (2.1%)	Passed	Omicron5
August	22055389	26,46	BA.5.1	893 (3.0%)	Passed	Omicron5
August	22055663	26,38	BE.1.1	531 (1.8%)	Passed	Omicron5
August	22056493	26,1	BE.1	971 (3.3%)	Passed	Omicron5
August	22056813	26,07	BA.5.1.2	1071 (3.6%)	Passed	Omicron5
August	22055360	26,06	BA.5.1	813 (2.7%)	Passed	Omicron5
August	22057288	26	BA.5.1.5	604 (2.0%)	Passed	Omicron5
August	22057890	25,93	BA.5.2	641 (2.1%)	Passed	Omicron5
August	22055335	25,85	BA.5.1	21 (0.1%)	Passed	Omicron5
August	22055376	25,62	BA.5.1	884 (3.0%)	Passed	Omicron5
August	22057660	25,62	BA.5.1.30	888 (3.0%)	Passed	Omicron5
August	22055357	25,55	BA.5.1	116 (0.4%)	Passed	Omicron5
August	22055669	25,23	BA.4	535 (1.8%)	Passed	Omicron4
August	22055339	25,18	BA.5.1.30	459 (1.5%)	Passed	Omicron5
August	22057135	25,16	BA.5.1	487 (1.6%)	Passed	Omicron5
August	22057991	24,86	BF.5	888 (3.0%)	Passed	Omicron5
August	22055331	24,75	BA.5.1	217 (0.7%)	Passed	Omicron5
August	22056706	24,51	BA.5.2.2	424 (1.4%)	Passed	Omicron5
August	22056213	24,49	BA.5.2	116 (0.4%)	Passed	Omicron5
August	22058130	24,48	BA.5.1	1212 (4.1%)	Passed	Omicron5
August	22057378	24,43	BA.5.2.1	563 (1.9%)	Passed	Omicron5
August	22055338	24,26	BA.5.1	574 (1.9%)	Passed	Omicron5
August	22055361	24,23	BA.5.1.35	562 (1.9%)	Passed	Omicron5
August	22056704	24,2	BA.5.1.35	828 (2.8%)	Passed	Omicron5
August	22057983	24	BA.5.9	232 (0.8%)	Passed	Omicron5
August	22055355	23,97	BA.5.1	552 (1.9%)	Passed	Omicron5
August	22058125	23,74	BA.5.1	1298 (4.4%)	Passed	Omicron5
August	22057650	23,59	BA.5.2.21	866 (2.9%)	Passed	Omicron5
August	22057025	23,57	BA.5.2.1	241 (0.8%)	Passed	Omicron5
August	22056337	23,51	BA.5.1	619 (2.1%)	Passed	Omicron5
August	22055940	23,26	BA.5	180 (0.6%)	Passed	Omicron5
August	22055381	23,23	BA.2.18	535 (1.8%)	Passed	Omicron2
August	22055408	23,18	BA.5.2.1	345 (1.2%)	Passed	Omicron5
August	22055343	23,01	BA.5.1.30	722 (2.4%)	Passed	Omicron5
August	22057385	23,01	BA.5.1	546 (1.8%)	Passed	Omicron5
August	22058432	22,87	BA.5.2.1	755 (2.5%)	Passed	Omicron5
August	22055365	22,76	BA.5.1.9	340 (1.1%)	Passed	Omicron5
August	22056354	22,67	BA.4.6	561 (1.9%)	Passed	Omicron4

August	22056499	22,66	BA.5.1.22	228 (0.8%)	Passed	Omicron5
August	22058325	22,61	BA.5.2	492 (1.7%)	Passed	Omicron5
August	22055334	22,54	BA.5	118 (0.4%)	Passed	Omicron5
August	22056339	22,5	BA.5.2	280 (0.9%)	Passed	Omicron5
August	22055347	22,49	BA.5.1	34 (0.1%)	Passed	Omicron5
August	22056220	22,48	BA.2.12.1	353 (1.2%)	Passed	Omicron2
August	22057388	22,47	BE.1.1	281 (0.9%)	Passed	Omicron5
August	22055659	22,46	BE.1.1	156 (0.5%)	Passed	Omicron5
August	22055415	22,39	BA.5.1	118 (0.4%)	Passed	Omicron5
August	22058329	22,39	BA.5.3	118 (0.4%)	Passed	Omicron5
August	22055939	22,38	BA.5.2.1	360 (1.2%)	Passed	Omicron5
August	22056218	22,2	BA.5.2.28	303 (1.0%)	Passed	Omicron5
August	22057044	22,19	BA.5.2.1	208 (0.7%)	Passed	Omicron5
August	22056808	22,18	BA.5.2.1	728 (2.4%)	Passed	Omicron5
August	22055662	21,94	BE.1.1	255 (0.9%)	Passed	Omicron5
August	22055364	21,91	BA.4	65 (0.2%)	Passed	Omicron4
August	22056815	21,86	BA.5.1.2	482 (1.6%)	Passed	Omicron5
August	22055327	21,79	BA.5.1.21	586 (2.0%)	Passed	Omicron5
August	22057019	21,72	BA.5.2.21	218 (0.7%)	Passed	Omicron5
August	22057268	21,69	BF.28	170 (0.6%)	Passed	Omicron5
August	22055667	21,68	BA.4.6	63 (0.2%)	Passed	Omicron4
August	22057850	21,64	BA.5.1.1	234 (0.8%)	Passed	Omicron5
August	22057853	21,59	BA.5.2	313 (1.0%)	Passed	Omicron5
August	22058322	21,49	BA.5.1	316 (1.1%)	Passed	Omicron5
August	22055668	21,35	BA.5.2.1	194 (0.7%)	Passed	Omicron5
August	22055395	21,33	BA.5.2.1	325 (1.1%)	Passed	Omicron5
August	22055938	21,18	BA.5.2	348 (1.2%)	Passed	Omicron5
August	22055375	20,89	BA.4	186 (0.6%)	Passed	Omicron4
August	22056916	20,85	BA.5.1.8	313 (1.0%)	Passed	Omicron5
August	22055350	20,75	BA.5.1	243 (0.8%)	Passed	Omicron5
August	22055855	20,75	BF.5	64 (0.2%)	Passed	Omicron5
August	22055419	20,71	BA.5.1.10	293 (1.0%)	Passed	Omicron5
August	22057129	20,69	BA.5.1	239 (0.8%)	Passed	Omicron5
August	22058430	20,68	BA.5.1.24	294 (1.0%)	Passed	Omicron5
August	22055660	20,57	BA.5.1	39 (0.1%)	Passed	Omicron5
August	22057888	20,52	BA.5.1.30	183 (0.6%)	Passed	Omicron5
August	22056492	20,44	BA.5.1	201 (0.7%)	Passed	Omicron5
August	22056335	20,41	BA.5.1	264 (0.9%)	Passed	Omicron5
August	22057396	20,4	BA.5.1	80 (0.3%)	Passed	Omicron5
August	22058438	20,37	BA.5.2.1	295 (1.0%)	Passed	Omicron5
August	22055384	20,35	BA.5.1	210 (0.7%)	Passed	Omicron5
August	22056624	20,3	BA.5.2.1	87 (0.3%)	Passed	Omicron5
August	22057265	20,26	BA.5.1	343 (1.1%)	Passed	Omicron5
August	22055411	20,16	BA.4.1.1	299 (1.0%)	Passed	Omicron4
August	22056908	20,12	BA.5.1	244 (0.8%)	Passed	Omicron5
August	22055860	20,09	BA.5.2	118 (0.4%)	Passed	Omicron5
August	22055933	20,08	BA.5	2283 (7.7%)	Failed	Omicron5
August	22055346	20,06	BA.5.2.1	142 (0.5%)	Passed	Omicron5
August	22056707	20,05	BA.5.2	254 (0.9%)	Passed	Omicron5
August	22055856	20,02	BA.5.1	130 (0.4%)	Passed	Omicron5
August	22055393	19,98	BA.5.1	569 (1.9%)	Passed	Omicron5
August	22056548	19,92	BA.5.2.1	118 (0.4%)	Passed	Omicron5
August	22055670	19,9	BA.4	71 (0.2%)	Passed	Omicron4
August	22056334	19,89	BA.5.2.1	278 (0.9%)	Passed	Omicron5
August	22057981	19,85	BE.1.1	110 (0.4%)	Passed	Omicron5
August	22057849	19,82	BE.1	18 (0.1%)	Passed	Omicron5
August	22057015	19,66	BA.4	136 (0.5%)	Passed	Omicron4
August	22056636	19,63	BF.7	118 (0.4%)	Passed	Omicron5
August	22056819	19,51	BA.5.1	412 (1.4%)	Passed	Omicron5
August	22058131	19,32	BA.5.1	1631 (5.5%)	Failed	Omicron5
August	22057380	19,29	BA.5.2.1	60 (0.2%)	Passed	Omicron5
August	22057124	19,27	BA.5.1.10	297 (1.0%)	Passed	Omicron5
August	22058330	19,17	BA.5.2.21	301 (1.0%)	Passed	Omicron5
August	22057375	19,11	BA.5.2.20	118 (0.4%)	Passed	Omicron5
August	22057022	19,07	BE.1.1	166 (0.6%)	Passed	Omicron5
August	22055359	19,02	BA.5.1	69 (0.2%)	Passed	Omicron5
August	22056620	18,99	BE.1.1.2	42 (0.1%)	Passed	Omicron5
August	22055344	18,98	BA.5.1	83 (0.3%)	Passed	Omicron5
August	22055946	18,97	BF.11.1	110 (0.4%)	Passed	Omicron5
August	22056705	18,89	BA.4.6	162 (0.5%)	Passed	Omicron4
August	22056214	18,88	BA.4	287 (1.0%)	Passed	Omicron4

August	22055326	18,84	BA.5.1	289 (1.0%)	Passed	Omicron5
August	22055370	18,75	BF.27	295 (1.0%)	Passed	Omicron5
August	22055373	18,71	BA.5.1	179 (0.6%)	Passed	Omicron5
August	22056628	18,7	BA.5.2.1	109 (0.4%)	Passed	Omicron5
August	22056330	18,64	BA.5.5	336 (1.1%)	Passed	Omicron5
August	22055853	18,59	BA.5.1	192 (0.6%)	Passed	Omicron5
August	22058427	18,55	BA.5.2.1	298 (1.0%)	Passed	Omicron5
August	22057036	18,51	BE.1.1	184 (0.6%)	Passed	Omicron5
August	22055358	18,49	BA.5.2.1	271 (0.9%)	Passed	Omicron5
August	22056708	18,49	BA.5.1	321 (1.1%)	Passed	Omicron5
August	22058128	18,42	BA.5.1.21	292 (1.0%)	Passed	Omicron5
August	22058132	18,32	BA.5.1	619 (2.1%)	Passed	Omicron5
August	22057014	18,3	BA.5.2	50 (0.2%)	Passed	Omicron5
August	22058429	18,27	BA.5.2	244 (0.8%)	Passed	Omicron5
August	22057371	18,24	BA.5.2.3	71 (0.2%)	Passed	Omicron5
August	22058134	18,23	BA.5.1.30	537 (1.8%)	Passed	Omicron5
August	22056621	17,73	BA.5.2	116 (0.4%)	Passed	Omicron5
August	22056623	17,73	BA.5.2.1	82 (0.3%)	Passed	Omicron5
August	22056907	17,55	BA.5.2.1	110 (0.4%)	Passed	Omicron5
August	22055329	17,54	BA.5.1.22	248 (0.8%)	Passed	Omicron5
August	22055657	17,53	BA.5.1	374 (1.3%)	Passed	Omicron5
August	22057274	17,52	BF.5	396 (1.3%)	Passed	Omicron5
August	22056703	17,42	BF.1	72 (0.2%)	Passed	Omicron5
August	22058328	17,34	BA.5.3	64 (0.2%)	Passed	Omicron5
August	22058133	17,31	BA.5.1	513 (1.7%)	Passed	Omicron5
August	22058323	17,25	BA.5.1	294 (1.0%)	Passed	Omicron5
August	22057123	17,21	BA.5.1.10	197 (0.7%)	Passed	Omicron5
August	22057275	17,21	BA.5.2.1	223 (0.7%)	Passed	Omicron5
August	22055858	17,15	BA.5.1.3	862 (2.9%)	Passed	Omicron5
August	22057279	17,14	BA.5.2	97 (0.3%)	Passed	Omicron5
August	22057989	17,14	BA.5.2.21	115 (0.4%)	Passed	Omicron5
August	22055382	17,08	BA.5.1	91 (0.3%)	Passed	Omicron5
August	22056340	17,08	BA.5.2.1	116 (0.4%)	Passed	Omicron5
August	22056215	17,05	BA.5.1	116 (0.4%)	Passed	Omicron5
August	22056702	17,03	BA.5.2.1	23 (0.1%)	Passed	Omicron5
August	22057980	17,02	BF.7	91 (0.3%)	Passed	Omicron5
August	22058327	16,98	BA.5.2.1	352 (1.2%)	Passed	Omicron5
August	22057894	16,9	BA.5.1	326 (1.1%)	Passed	Omicron5
August	22057112	16,73	BA.5.1	118 (0.4%)	Passed	Omicron5
August	22056331	16,59	BF.1	26 (0.1%)	Passed	Omicron5
August	22057847	16,58	BA.5.2	152 (0.5%)	Passed	Omicron5
August	22056629	16,42	BA.5.1	117 (0.4%)	Passed	Omicron5
August	22055418	16,41	BA.5.1	150 (0.5%)	Passed	Omicron5
August	22056516	16,41	BA.5.2.1	135 (0.5%)	Passed	Omicron5
August	22055345	16,4	BA.5.2.21	90 (0.3%)	Passed	Omicron5
August	22057291	16,3	BA.5.1.23	14 (0.0%)	Passed	Omicron5
August	22058129	16,19	XAZ	15 (0.1%)	Passed	Ricombinante
August	22055366	16,08	BA.5.1.9	80 (0.3%)	Passed	Omicron5
August	22057017	16,06	BA.5.1.30	68 (0.2%)	Passed	Omicron5
August	22058321	16,01	BA.2	310 (1.0%)	Passed	Omicron5
August	22056919	15,92	BA.5.1.30	42 (0.1%)	Passed	Omicron5
August	22058326	15,9	BA.5.2	219 (0.7%)	Passed	Omicron5
August	22055330	15,85	BA.5.1	73 (0.2%)	Passed	Omicron5
August	22057647	15,85	BA.5.1	23 (0.1%)	Passed	Omicron5
August	22055351	15,76	BA.5.1	101 (0.3%)	Passed	Omicron5
August	22058127	15,75	BA.5.1.30	221 (0.7%)	Passed	Omicron5
August	22057640	15,69	BE.1.1	86 (0.3%)	Passed	Omicron5
August	22056710	15,63	BA.5.2.1	74 (0.2%)	Passed	Omicron5
August	22057884	15,6	BA.5.2.1	69 (0.2%)	Passed	Omicron5
August	22057372	15,57	BA.5.1.23	65 (0.2%)	Passed	Omicron5
August	22057639	15,54	BA.5.1	181 (0.6%)	Passed	Omicron5
August	22056343	15,51	BA.5.1	70 (0.2%)	Passed	Omicron5
August	22055369	15,43	BA.4	66 (0.2%)	Passed	Omicron4
August	22057648	15,4	BA.5.3.2	22 (0.1%)	Passed	Omicron5
August	22056630	15,35	BA.5.2.1	76 (0.3%)	Passed	Omicron5
August	22056711	15,33	BA.5.2.1	15 (0.1%)	Passed	Omicron5
August	22055372	15,32	BA.5.1	118 (0.4%)	Passed	Omicron5
August	22056217	15,27	BA.5.2	118 (0.4%)	Passed	Omicron5
August	22057642	15,24	BF.5	61 (0.2%)	Passed	Omicron5
August	22057115	15,21	BA.5.2.9	69 (0.2%)	Passed	Omicron5
August	22058433	15,19	BA.5.1.5	116 (0.4%)	Passed	Omicron5

August	22056906	15,04	BA.5.2.1	68 (0.2%)	Passed	Omicron5
August	22057285	15,03	BF.7	75 (0.3%)	Passed	Omicron5
August	22057114	15,02	BA.5.2	65 (0.2%)	Passed	Omicron5
August	22056632	14,9	BE.1.1	73 (0.2%)	Passed	Omicron5
August	22057656	14,85	BA.5.2.1	26 (0.1%)	Passed	Omicron5
August	22058431	14,76	BE.1.1	114 (0.4%)	Passed	Omicron5
August	22056338	14,75	BA.5.2	35 (0.1%)	Passed	Omicron5
August	22056219	14,73	BA.5.2.1	115 (0.4%)	Passed	Omicron5
August	22055340	14,72	BA.5.2.1	334 (1.1%)	Passed	Omicron5
August	22056915	14,71	BA.5.10	19 (0.1%)	Passed	Omicron5
August	22056904	14,49	BE.4	76 (0.3%)	Passed	Omicron5
August	22056709	14,35	BA.5.2	108 (0.4%)	Passed	Omicron5
August	22057374	14,29	BA.5.1.23	46 (0.2%)	Passed	Omicron5
August	22055328	14,1	BA.5.1.22	68 (0.2%)	Passed	Omicron5
August	22056911	14,05	BA.4	118 (0.4%)	Passed	Omicron4
August	22056495	14,03	BE.4	33 (0.1%)	Passed	Omicron5
August	22057979	13,99	BA.5.2	38 (0.1%)	Passed	Omicron5
August	22055380	13,88	BA.5.1	68 (0.2%)	Passed	Omicron5
August	22056622	13,65	BE.1	51 (0.2%)	Passed	Omicron5
August	22056216	13,62	BA.5.1.23	82 (0.3%)	Passed	Omicron5
August	22055388	13,6	BA.5.1.30	30 (0.1%)	Passed	Omicron5
August	22055383	13,57	BA.5.1	78 (0.3%)	Passed	Omicron5
August	22058126	13,54	BA.5.2.9	120 (0.4%)	Passed	Omicron5
August	22055396	13,5	BA.5.2.1	68 (0.2%)	Passed	Omicron5
August	22057264	13,28	BA.5.1	46 (0.2%)	Passed	Omicron5
August	22057016	13,24	BA.4	68 (0.2%)	Passed	Omicron4
August	22055394	13,19	BA.5.1	293 (1.0%)	Passed	Omicron5
August	22055349	13,02	BA.5.1	68 (0.2%)	Passed	Omicron5
August	22055404	13,02	BA.5.1	20 (0.1%)	Passed	Omicron5
August	22057985	12,91	BA.5.2	28 (0.1%)	Passed	Omicron5
August	22056547	12,78	BA.5.2.1	118 (0.4%)	Passed	Omicron5
August	22057644	12,71	BA.5.2	118 (0.4%)	Passed	Omicron5
August	22056810	12,41	BA.5.2.1	69 (0.2%)	Passed	Omicron5
August	22056924	12,41	BA.5.1	70 (0.2%)	Passed	Omicron5
August	22055342	12,34	BA.5.1	70 (0.2%)	Passed	Omicron5
August	22055336	12,12	BA.5.1	62 (0.2%)	Passed	Omicron5
August	22055368	12,11	BA.5.1.1	68 (0.2%)	Passed	Omicron5
August	22057892	11,87	XAZ	70 (0.2%)	Passed	Ricombinante

Supplementary Table S4

Month	Sample	Valore CT	Lineage	N_consensus	QC_status	Variante
September	22059664	29,99	BA.5.1	1470 (4.9%)	Passed	Omicron5
September	22059546	29,56	BA.5.1	1595 (5.4%)	Failed	Omicron5
September	22058990	29,32	BA.5.2	2236 (7.5%)	Failed	Omicron5
September	22058918	29,3	BA.5.1	2152 (7.2%)	Failed	Omicron5
September	22059085	28,77	BA.5	2137 (7.3%)	Failed	Omicron5
September	22058987	28,59	BA.5.2.21	1737 (5.8%)	Failed	Omicron5
September	22058824	28,47	BA.5.2	2663 (9.0%)	Failed	Omicron5
September	22058923	28,39	BA.5.1	1455 (4.9%)	Passed	Omicron5
September	22058929	28,38	BA.5	2486 (8.4%)	Failed	Omicron5
September	22058511	28,33	BA.5.1	1820 (6.1%)	Failed	Omicron5
September	22059553	28,32	BA.5.1	1233 (4.1%)	Passed	Omicron5
September	22059667	28,06	BA.5.2.1	1261 (4.2%)	Passed	Omicron5
September	22058859	27,96	BA.5.1	2751 (9.2%)	Failed	Omicron5
September	22058989	27,96	BA.5.1	4804 (16.1%)	Failed	Omicron5
September	22059091	27,94	BA.5.2	988 (3.3%)	Passed	Omicron5
September	22060107	27,94	BA.2	2433 (8.2%)	Failed	Omicron2
September	22059094	27,42	BA.5.2.33	891 (3.0%)	Passed	Omicron5
September	22059333	27,41	BA.5.1.30	950 (3.2%)	Passed	Omicron5
September	22060007	27,4	BA.4	943 (3.2%)	Passed	Omicron4
September	22058820	27,29	BA.5.1.22	1624 (5.4%)	Failed	Omicron5
September	22059339	27,21	BA.5.2	849 (2.8%)	Passed	Omicron5
September	22059554	27,21	BA.5.1.24	1104 (3.7%)	Passed	Omicron5
September	22059923	27,09	BE.1.1	763 (2.6%)	Passed	Omicron5
September	22058832	26,94	BF.28	1128 (3.8%)	Passed	Omicron5
September	22060212	26,84	BA.5.2.1	312 (1.0%)	Passed	Omicron5
September	22060004	26,79	BA.4	1079 (3.6%)	Passed	Omicron4
September	22060016	26,74	BA.5.2.1	1297 (4.4%)	Passed	Omicron5
September	22059107	26,65	BA.5.1.30	2012 (6.8%)	Failed	Omicron5
September	22059596	26,64	BA.5.1	868 (2.9%)	Passed	Omicron5
September	22058837	26,46	BA.5.2.1	1417 (4.8%)	Passed	Omicron5
September	22059114	26,36	BA.5.1.30	1426 (4.8%)	Passed	Omicron5
September	22058822	26,13	BA.5.1	1143 (3.8%)	Passed	Omicron5
September	22059097	26,12	BA.2	190 (0.6%)	Passed	Omicron2
September	22058919	25,8	BA.2	655 (2.2%)	Passed	Omicron2
September	22058830	25,79	BA.5.2.26	1563 (5.2%)	Failed	Omicron5
September	22058571	25,71	BA.5.2.1	1377 (4.6%)	Passed	Omicron5
September	22059762	25,65	BA.5.2	602 (2.0%)	Passed	Omicron5
September	22059669	25,49	BE.1.1	913 (3.1%)	Passed	Omicron5
September	22058860	25,26	BA.5.2.1	1629 (5.5%)	Failed	Omicron5
September	22059765	25,23	BA.5.1	3070 (10.3%)	Failed	Omicron5
September	22058985	25,2	BA.5.1.30	575 (1.9%)	Passed	Omicron5
September	22059550	25,2	BA.5	2481 (8.3%)	Failed	Omicron5
September	22058833	24,92	BA.4.4	1401 (4.7%)	Passed	Omicron4
September	22058590	24,76	BA.5.1	927 (3.1%)	Passed	Omicron5
September	22059665	24,4	BA.5.2.1	845 (2.8%)	Passed	Omicron5
September	22058836	24,37	BA.5.6	961 (3.2%)	Passed	Omicron5
September	22059332	24,37	BA.4.1	588 (2.0%)	Passed	Omicron4
September	22058506	24,34	BA.5.2.1	802 (2.7%)	Passed	Omicron5
September	22059102	24,32	BA.5.2	732 (2.5%)	Passed	Omicron5
September	22059965	24,24	BF.5	345 (1.2%)	Passed	Omicron5
September	22059330	24,21	BA.5.1.30	647 (2.2%)	Passed	Omicron5
September	22059988	24,13	BF.10	259 (0.9%)	Passed	Omicron5
September	22058718	24,1	BA.5.2.1	687 (2.3%)	Passed	Omicron5
September	22059924	24	BA.5.2.20	317 (1.1%)	Passed	Omicron5
September	22059326	23,83	BA.5.2.1	627 (2.1%)	Passed	Omicron5
September	22058862	23,5	BA.5.2.1	524 (1.8%)	Passed	Omicron5
September	22058569	23,43	BA.5.1	827 (2.8%)	Passed	Omicron5
September	22058717	23,26	BA.5.1.10	705 (2.4%)	Passed	Omicron5
September	22059764	23,26	BA.5.1	601 (2.0%)	Passed	Omicron5

September	22060219	23,23	BA.5.2	413 (1.4%)	Passed	Omicron5
September	22058928	23,22	BA.5.6	576 (1.9%)	Passed	Omicron5
September	22058721	23,07	BA.5.1	579 (1.9%)	Passed	Omicron5
September	22059670	22,99	BA.5.2.1	264 (0.9%)	Passed	Omicron5
September	22059852	22,94	BA.5.1	345 (1.2%)	Passed	Omicron5
September	22059106	22,81	BA.5.1.30	519 (1.7%)	Passed	Omicron5
September	22058857	22,68	BA.5.2.1	472 (1.6%)	Passed	Omicron5
September	22059324	22,64	BA.5.1	268 (0.9%)	Passed	Omicron5
September	22059551	22,57	BA.5.1.23	532 (1.8%)	Passed	Omicron5
September	22059092	22,56	BA.5.1	70 (0.2%)	Passed	Omicron5
September	22060313	22,51	BF.7	460 (1.5%)	Passed	Omicron5
September	22058826	22,44	BA.5.2	456 (1.5%)	Passed	Omicron5
September	22060020	22,42	BA.4.6	565 (1.9%)	Passed	Omicron4
September	22059763	22,3	BA.5.2	164 (0.5%)	Passed	Omicron5
September	22060101	21,99	BA.5.1.23	392 (1.3%)	Passed	Omicron5
September	22059661	21,91	BA.5.2.1	201 (0.7%)	Passed	Omicron5
September	22059934	21,75	BA.5.2.16	448 (1.5%)	Passed	Omicron5
September	22059087	21,47	BA.5.1	226 (0.8%)	Passed	Omicron5
September	22058512	21,44	BA.5.1	572 (1.9%)	Passed	Omicron5
September	22060312	21,41	BA.5.2	149 (0.5%)	Passed	Omicron5
September	22060099	21,19	BA.5.1.10	10 (0.0%)	Passed	Omicron5
September	22059668	21,02	BA.5.2.21	208 (0.7%)	Passed	Omicron5
September	22058984	21,01	BA.5	2115 (7.1%)	Failed	Omicron5
September	22058827	20,93	BA.5.1.30	303 (1.0%)	Passed	Omicron5
September	22059325	20,92	BA.5.2.1	293 (1.0%)	Passed	Omicron5
September	22058515	20,79	BA.5.1	492 (1.6%)	Passed	Omicron5
September	22059922	20,77	BA.4.6	105 (0.4%)	Passed	Omicron4
September	22058941	20,66	BA.5.2.1	284 (1.0%)	Passed	Omicron5
September	22058943	20,59	BA.4.4	589 (2.0%)	Passed	Omicron4
September	22058821	20,54	BA.5.2	303 (1.0%)	Passed	Omicron5
September	22059086	20,45	BA.5.1	300 (1.0%)	Passed	Omicron5
September	22060010	20,38	BA.5.2.20	115 (0.4%)	Passed	Omicron5
September	22058722	20,34	BM.1.1.3	294 (1.0%)	Passed	Omicron2
September	22058828	20,26	BA.5.1.30	306 (1.0%)	Passed	Omicron5
September	22060008	20,22	BA.5.1	233 (0.8%)	Passed	Omicron5
September	22058863	20,18	BE.1	301 (1.0%)	Passed	Omicron5
September	22059095	20,17	BA.5.1	562 (1.9%)	Passed	Omicron5
September	22059100	20,07	BA.5.2.1	294 (1.0%)	Passed	Omicron5
September	22059590	19,97	BA.5.1	376 (1.3%)	Passed	Omicron5
September	22058861	19,95	BA.5.2.1	395 (1.3%)	Passed	Omicron5
September	22059593	19,92	BA.5.2.1	248 (0.8%)	Passed	Omicron5
September	22058720	19,75	BA.5.2.1	349 (1.2%)	Passed	Omicron5
September	22060213	19,73	BA.5.2	191 (0.6%)	Passed	Omicron5
September	22059110	19,65	BA.4.1	307 (1.0%)	Passed	Omicron4
September	22058510	19,47	BA.5.1.10	276 (0.9%)	Passed	Omicron5
September	22059925	19,46	BA.5.1	33 (0.1%)	Passed	Omicron5
September	22059592	19,39	BA.5.2.1	83 (0.3%)	Passed	Omicron5
September	22058823	19,38	BA.5.1.5	344 (1.2%)	Passed	Omicron5
September	22059594	19,33	BE.1	278 (0.9%)	Passed	Omicron5
September	22058834	19,28	BA.5.6	292 (1.0%)	Passed	Omicron5
September	22059101	19,27	BA.5.1	582 (2.0%)	Passed	Omicron5
September	22058587	19,01	BA.5.1.2	308 (1.0%)	Passed	Omicron5
September	22059104	19,01	BA.5.2	259 (0.9%)	Passed	Omicron5
September	22059598	18,99	BF.5	36 (0.1%)	Passed	Omicron5
September	22058831	18,96	CG.1	294 (1.0%)	Passed	Omicron5
September	22059850	18,61	BA.5.2.9	120 (0.4%)	Passed	Omicron5
September	22059549	18,6	BA.5.1.10	128 (0.4%)	Passed	Omicron5
September	22058568	18,58	BA.5.2.1	297 (1.0%)	Passed	Omicron5
September	22060330	18,52	BA.5.2.16	31 (0.1%)	Passed	Omicron5
September	22058992	18,48	BA.5.1.1	290 (1.0%)	Passed	Omicron5
September	22059552	18,26	BA.5.1.23	135 (0.5%)	Passed	Omicron5

September	22059096	18,18	BA.5.1	609 (2.0%)	Passed	Omicron5
September	22058927	18,16	BA.4.6.5	290 (1.0%)	Passed	Omicron4
September	22060103	18,11	BF.7.21	66 (0.2%)	Passed	Omicron5
September	22059591	18,02	BA.5.2.1	17 (0.1%)	Passed	Omicron5
September	22059663	17,98	BA.5.2	77 (0.3%)	Passed	Omicron5
September	22059111	17,87	BA.4.1	315 (1.1%)	Passed	Omicron4
September	22059336	17,84	BA.5.2.1	101 (0.3%)	Passed	Omicron5
September	22059666	17,83	BA.5.2.12	75 (0.3%)	Passed	Omicron5
September	22059964	17,75	BA.4.6	248 (0.8%)	Passed	Omicron4
September	22059595	17,71	BA.5.1	546 (1.8%)	Passed	Omicron5
September	22059547	17,67	BA.4.6	272 (0.9%)	Passed	Omicron4
September	22059103	17,61	BA.5.2.33	73 (0.2%)	Passed	Omicron5
September	22060100	17,56	BF.5	144 (0.5%)	Passed	Omicron5
September	22058570	17,54	BA.5.2.1	181 (0.6%)	Passed	Omicron5
September	22059933	17,52	BA.5.2.16	39 (0.1%)	Passed	Omicron5
September	22059597	17,46	BA.5.2.2	46 (0.2%)	Passed	Omicron5
September	22059966	17,44	BA.5.1	146 (0.5%)	Passed	Omicron5
September	22060316	17,38	BA.5.1.30	65 (0.2%)	Passed	Omicron5
September	22058991	17,19	BA.5.2	85 (0.3%)	Passed	Omicron5
September	22058838	17,14	BF.1	294 (1.0%)	Passed	Omicron5
September	22058858	17,12	BA.5.1	467 (1.6%)	Passed	Omicron5
September	22058835	16,82	BA.5.6	116 (0.4%)	Passed	Omicron5
September	22060248	16,81	BA.5.1	338 (1.1%)	Passed	Omicron5
September	22058925	16,76	BE.1.1	63 (0.2%)	Passed	Omicron5
September	22058508	16,68	BA.5.1	448 (1.5%)	Passed	Omicron5
September	22058589	16,67	BA.5.1	234 (0.8%)	Passed	Omicron5
September	22059783	16,66	BA.5.2.20	41 (0.1%)	Passed	Omicron5
September	22059112	16,64	BA.4.1	298 (1.0%)	Passed	Omicron4
September	22058993	16,62	BA.5.1	76 (0.3%)	Passed	Omicron5
September	22059851	16,55	BA.5.1.22	53 (0.2%)	Passed	Omicron5
September	22059089	16,54	BA.5.2.1	116 (0.4%)	Passed	Omicron5
September	22059589	16,54	BA.5.1	294 (1.0%)	Passed	Omicron5
September	22060246	16,4	BE.1.1	68 (0.2%)	Passed	Omicron5
September	22059088	16,33	BA.5.1	33 (0.1%)	Passed	Omicron5
September	22060003	16,24	BA.4.6	65 (0.2%)	Passed	Omicron4
September	22060250	16,07	BE.1	86 (0.3%)	Passed	Omicron5
September	22060005	16,05	BA.5.1	310 (1.0%)	Passed	Omicron5
September	22059662	16,03	BA.5.2.1	73 (0.2%)	Passed	Omicron5
September	22059105	16	BA.5.1	78 (0.3%)	Passed	Omicron5
September	22060337	15,96	BA.5.1	72 (0.2%)	Passed	Omicron5
September	22060009	15,85	BA.5.1	68 (0.2%)	Passed	Omicron5
September	22060309	15,81	BE.1.1	72 (0.2%)	Passed	Omicron5
September	22059099	15,65	BA.4.1	63 (0.2%)	Passed	Omicron4
September	22060307	15,62	BE.1.1	68 (0.2%)	Passed	Omicron5
September	22058986	15,59	Unassigned	20663 (69.4%)	Failed	Omicron
September	22060249	15,57	BA.5.1	1041 (3.5%)	Passed	Omicron5
September	22060098	15,45	BA.4.6	89 (0.3%)	Passed	Omicron4
September	22058719	15,38	BA.5.2	206 (0.7%)	Passed	Omicron5
September	22058819	15,33	BA.5.1.22	68 (0.2%)	Passed	Omicron5
September	22059108	15,22	BA.5.1	76 (0.3%)	Passed	Omicron5
September	22059098	15,18	BA.5.1.30	27 (0.1%)	Passed	Omicron5
September	22060105	15,14	BA.5.2.1	75 (0.3%)	Passed	Omicron5
September	22059960	15,08	BA.5.1	70 (0.2%)	Passed	Omicron5
September	22059548	15,06	BA.5.1.10	113 (0.4%)	Passed	Omicron5
September	22058988	15,05	BF.7	75 (0.3%)	Passed	Omicron5
September	22060306	15	BA.5.1.10	17 (0.1%)	Passed	Omicron5
September	22059854	14,95	BE.1	68 (0.2%)	Passed	Omicron5
September	22059776	14,82	BA.5.2.20	46 (0.2%)	Passed	Omicron5
September	22059963	14,7	BA.5.2	118 (0.4%)	Passed	Omicron5
September	22060247	14,61	BE.1.1	66 (0.2%)	Passed	Omicron5
September	22059967	14,41	BE.1.1	73 (0.2%)	Passed	Omicron5

September	22058513	14,28	BA.5.1	523 (1.8%)	Passed	Omicron5
September	22059939	14,09	BA.5.2.1	71 (0.2%)	Passed	Omicron5
September	22060251	13,92	BA.5.2	68 (0.2%)	Passed	Omicron5
September	22060104	13,89	BF.7.21	68 (0.2%)	Passed	Omicron5
September	22059328	13,2	BA.5.2.18	67 (0.2%)	Passed	Omicron5
September	22059772	13,19	BA.4.6	58 (0.2%)	Passed	Omicron4
September	22058507	13,17	BA.5.1	439 (1.5%)	Passed	Omicron5
September	22060218	13,15	BA.5.1	283 (0.9%)	Passed	Omicron5
September	22058514	13,09	BA.5.2.1	17 (0.1%)	Passed	Omicron5
September	22058591	12,94	BA.5.2	78 (0.3%)	Passed	Omicron5
September	22060106	12,91	BA.4.6	69 (0.2%)	Passed	Omicron4
September	22059761	12,83	BA.5.2	39 (0.1%)	Passed	Omicron5
September	22059329	12,79	BA.5.1	22 (0.1%)	Passed	Omicron5
September	22058920	12,71	BE.1.1	75 (0.3%)	Passed	Omicron5
September	22058572	12,52	BA.5.2.16	15 (0.1%)	Passed	Omicron5
September	22060102	12,5	BA.5.2	16 (0.1%)	Passed	Omicron5
September	22058856	12,43	BA.5.2	66 (0.2%)	Passed	Omicron5
September	22059770	12,2	BE.1.1	70 (0.2%)	Passed	Omicron5
September	22059853	11,37	BE.1	68 (0.2%)	Passed	Omicron5
September	22058509	10,99	BA.5.1.10	70 (0.2%)	Passed	Omicron5

Supplementary Table S5

Month	Sample	Valore CT	Lineage	N_consensus	QC_status	Variante
October	22062430	29,92	BQ.1.1	8259 (28.0%)	Failed	Omicron5
October	22060749	29,66	BA.5.1.22	581 (2.0%)	Passed	Omicron5
October	22060879	29,53	BA.5.1	546 (1.8%)	Passed	Omicron5
October	22062896	29,43	BA.5.2.1	109 (0.4%)	Passed	Omicron5
October	22062895	29,36	BF.11	8605 (29.1%)	Failed	Omicron5
October	22062440	29,18	Unassigned	9400 (31.8%)	Failed	Omicron
October	22060750	28,59	BA.5.1.30	601 (2.0%)	Passed	Omicron5
October	22064175	28,24	BA.5.1	1578 (5.3%)	Failed	Omicron5
October	22060753	27,52	BE.1	489 (1.6%)	Passed	Omicron5
October	22062050	27,3	BA.5.1	1363 (4.6%)	Passed	Omicron5
October	22060883	27,03	BA.5.1	523 (1.8%)	Passed	Omicron5
October	22060886	26,44	BF.4	368 (1.2%)	Passed	Omicron5
October	22060880	26,42	BA.5.2	307 (1.0%)	Passed	Omicron5
October	22062052	26,28	BF.3	1725 (5.8%)	Failed	Omicron5
October	22064446	26,19	BA.5.2.18	950 (3.2%)	Passed	Omicron5
October	22062057	25,88	BF.7.4.2	1143 (3.8%)	Passed	Omicron5
October	22061344	25,84	BE.1.2	2040 (6.8%)	Failed	Omicron5
October	22061861	25,83	BF.7.9	1078 (3.6%)	Passed	Omicron5
October	22063133	25,78	BA.5.2.6	3232 (10.9%)	Failed	Omicron5
October	22061508	25,72	BQ.1.13	1246 (4.2%)	Passed	Omicron5
October	22064231	25,55	XBB	662 (2.2%)	Passed	Omicron2
October	22061504	25,13	XBB	1436 (4.8%)	Passed	Omicron2
October	22064177	24,91	XBB	650 (2.2%)	Passed	Omicron2
October	22061083	24,83	BA.5.1.10	1496 (5.0%)	Failed	Omicron5
October	22060951	24,74	BE.1	1222 (4.1%)	Passed	Omicron5
October	22062051	24,74	BA.5.2.1	693 (2.3%)	Passed	Omicron5
October	22062595	24,69	BF.7	341 (1.1%)	Passed	Omicron5
October	22061094	24,57	BF.7	1255 (4.2%)	Passed	Omicron5
October	22064146	24,55	BQ.1	226 (0.8%)	Passed	Omicron5
October	22061415	24,5	BA.5.1	1273 (4.3%)	Passed	Omicron5
October	22061347	24,37	BQ.1	1157 (3.9%)	Passed	Omicron5
October	22061048	24,29	BQ.1.10	1241 (4.2%)	Passed	Omicron5
October	22062596	24,19	BF.7	535 (1.8%)	Passed	Omicron5
October	22062898	24,13	BA.5.1.5	2792 (9.4%)	Failed	Omicron5
October	22061089	24,1	XBB	1090 (3.7%)	Passed	Omicron2
October	22063815	24,03	BE.1	118 (0.4%)	Passed	Omicron5
October	22061343	23,96	BE.1.2	1180 (4.0%)	Passed	Omicron5
October	22061159	23,8	BA.5.5	1425 (4.8%)	Passed	Omicron5
October	22060754	23,62	BQ.1	220 (0.7%)	Passed	Omicron5
October	22062593	23,53	BE.4	473 (1.6%)	Passed	Omicron5
October	22064216	23,5	BA.5	1508 (5.1%)	Failed	Omicron5
October	22062794	23,49	Unassigned	10965 (36.8%)	Failed	Omicron
October	22062422	23,44	BE.1.1.2	116 (0.4%)	Passed	Omicron5
October	22061189	23,42	BA.5.1.10	1782 (6.0%)	Failed	Omicron5
October	22061501	23,4	BF.7.8	795 (2.7%)	Passed	Omicron5
October	22062449	23,35	BA.5.2.1	1243 (4.2%)	Passed	Omicron5
October	22063125	23,31	BA.5.1	1945 (6.5%)	Failed	Omicron5
October	22061509	23,22	BF.7.23	817 (2.7%)	Passed	Omicron5
October	22062787	23,16	XBB	331 (1.1%)	Passed	Ricombinante
October	22061081	23,13	BF.5	1245 (4.2%)	Passed	Omicron5
October	22061365	23,13	BE.1	1109 (3.7%)	Passed	Omicron5
October	22061047	23,07	BA.5.2	1445 (4.8%)	Passed	Omicron5
October	22061864	22,98	BF.7.9	273 (0.9%)	Passed	Omicron5
October	22062061	22,89	BA.5.1.5	322 (1.1%)	Passed	Omicron5
October	22062903	22,58	BF.21	1187 (4.0%)	Passed	Omicron5
October	22063322	22,53	BQ.1.1	698 (2.3%)	Passed	Omicron5
October	22061416	22,48	BQ.1.1	724 (2.4%)	Passed	Omicron5
October	22062453	22,46	BA.5.1.30	1056 (3.6%)	Passed	Omicron5
October	22063348	22,34	BQ.1.1	1428 (4.8%)	Passed	Omicron5
October	22061413	22,32	BF.7.4.2	314 (1.1%)	Passed	Omicron5
October	22062427	22,28	BA.5.2	890 (3.0%)	Passed	Omicron5
October	22061411	22,08	BF.14	804 (2.7%)	Passed	Omicron5
October	22061113	21,94	BQ.1.13	870 (2.9%)	Passed	Omicron5
October	22064186	21,89	BQ.1.1.11	125 (0.4%)	Passed	Omicron5
October	22060885	21,82	BF.4	271 (0.9%)	Passed	Omicron5
October	22061866	21,8	BA.5.2.1	177 (0.6%)	Passed	Omicron5
October	22061412	21,73	XBB.1.4	386 (1.3%)	Passed	Ricombinante
October	22061593	21,7	BA.5.1	629 (2.1%)	Passed	Omicron5
October	22063131	21,7	BQ.1.10	1227 (4.1%)	Passed	Omicron5
October	22062900	21,68	BN.3.1	1546 (5.2%)	Failed	Omicron2
October	22060770	21,56	BA.5.1	229 (0.8%)	Passed	Omicron5
October	22061346	21,44	BQ.1.2	847 (2.8%)	Passed	Omicron5
October	22064445	21,44	BA.5.2	508 (1.7%)	Passed	Omicron5
October	22062054	21,37	BQ.1.10	357 (1.2%)	Passed	Omicron5
October	22062901	21,31	BA.5.1.30	1114 (3.7%)	Passed	Omicron5
October	22060755	21,3	BF.7.6	265 (0.9%)	Passed	Omicron5
October	22061096	21,3	BA.5.2.38	478 (1.6%)	Passed	Omicron5
October	22061592	21,25	BA.5.1	755 (2.5%)	Passed	Omicron5
October	22060948	21,15	BF.39	791 (2.7%)	Passed	Omicron5
October	22061351	21,06	BE.4.1	536 (1.8%)	Passed	Omicron5
October	22061505	20,89	BF.10	513 (1.7%)	Passed	Omicron5
October	22063326	20,86	BF.7	316 (1.1%)	Passed	Omicron5
October	22061500	20,79	BA.5.1	691 (2.3%)	Passed	Omicron5
October	22061469	20,74	BA.5.2.20	384 (1.3%)	Passed	Omicron5
October	22061865	20,7	BA.5.2	74 (0.2%)	Passed	Omicron5
October	22064181	20,58	BQ.1	272 (0.9%)	Passed	Omicron5
October	22061506	20,55	BE.1	435 (1.5%)	Passed	Omicron5
October	22063823	20,48	BA.5.1.23	290 (1.0%)	Passed	Omicron5
October	22060748	20,46	BF.3	4295 (14.4%)	Failed	Omicron5
October	22060949	20,43	BF.11	470 (1.6%)	Passed	Omicron5
October	22061594	20,15	BA.5.1	554 (1.9%)	Passed	Omicron5
October	22061862	20,15	BA.5	102 (0.3%)	Passed	Omicron5
October	22062417	20,12	BQ.1.1	299 (1.0%)	Passed	Omicron5
October	22063349	20,02	BA.5.2	109 (0.4%)	Passed	Omicron5
October	22061049	19,97	BA.4.6	491 (1.6%)	Passed	Omicron4
October	22063329	19,83	XBB.1	117 (0.4%)	Passed	Ricombinante
October	22063338	19,79	BQ.1.1	267 (0.9%)	Passed	Omicron5
October	22061161	19,56	BF.3	697 (2.3%)	Passed	Omicron5

October	22064440	19,53	BF.7.21	300 (1.0%)	Passed	Omicron5
October	22063130	19,49	BA.5.2.1	113 (0.4%)	Passed	Omicron5
October	22061163	19,47	BA.5.1	238 (0.8%)	Passed	Omicron5
October	22063339	19,46	BQ.1.1	106 (0.4%)	Passed	Omicron5
October	22064223	19,34	BQ.1.10	177 (0.6%)	Passed	Omicron5
October	22064198	19,23	BA.5.2	4387 (14.7%)	Failed	Omicron5
October	22063331	19,17	BQ.1.2	117 (0.4%)	Passed	Omicron5
October	22061596	19,14	BA.2.75.2	271 (0.9%)	Passed	Omicron2
October	22063817	19,09	BA.5.2.1	56 (0.2%)	Passed	Omicron5
October	22064165	19,05	Unassigned	9481 (31.8%)	Failed	Omicron
October	22064182	18,95	BA.5.2	250 (0.8%)	Passed	Omicron5
October	22061088	18,83	BQ.1.10	483 (1.6%)	Passed	Omicron5
October	22061868	18,83	BA.5.2	83 (0.3%)	Passed	Omicron5
October	22061863	18,81	BA.5	74 (0.2%)	Passed	Omicron5
October	22061591	18,74	BQ.1	501 (1.7%)	Passed	Omicron5
October	22064441	18,71	BF.3	262 (0.9%)	Passed	Omicron5
October	22060944	18,64	BA.5.2	322 (1.1%)	Passed	Omicron5
October	22062902	18,56	BL.2	113 (0.4%)	Passed	Omicron2
October	22061053	18,51	BA.5.2	300 (1.0%)	Passed	Omicron5
October	22064180	18,5	BA.5.2.58	22 (0.1%)	Passed	Omicron5
October	22062435	18,37	BF.7	35 (0.1%)	Passed	Omicron5
October	22064238	18,31	BQ.1.1.4	70 (0.2%)	Passed	Omicron5
October	22061160	18,28	BA.5.5	174 (0.6%)	Passed	Omicron5
October	22063814	18,24	BQ.1.1.5	20 (0.1%)	Passed	Omicron5
October	22061595	18,23	BF.5	368 (1.2%)	Passed	Omicron5
October	22064444	18,17	BF.7.10	7633 (25.9%)	Failed	Omicron5
October	22062789	18,08	BA.5.1	442 (1.5%)	Passed	Omicron5
October	22062418	18,01	BF.7.5	77 (0.3%)	Passed	Omicron5
October	22062790	18	BA.5.2	118 (0.4%)	Passed	Omicron5
October	22061867	17,98	BA.5.2	120 (0.4%)	Passed	Omicron5
October	22061045	17,95	BE.1	221 (0.7%)	Passed	Omicron5
October	22060756	17,83	Unassigned	10164 (34.1%)	Failed	Omicron
October	22064174	17,83	BQ.1.15	275 (0.9%)	Passed	Omicron5
October	22063126	17,62	XBB.1.4	118 (0.4%)	Passed	Ricombinante
October	22063821	17,46	BA.5.1	118 (0.4%)	Passed	Omicron5
October	22061051	17,45	BA.4.6	294 (1.0%)	Passed	Omicron4
October	22061600	17,38	BA.5.1.23	291 (1.0%)	Passed	Omicron5
October	22064442	17,24	BQ.1.1	300 (1.0%)	Passed	Omicron5
October	22063818	16,97	BQ.1.1	64 (0.2%)	Passed	Omicron5
October	22061085	16,93	BF.11	84 (0.3%)	Passed	Omicron5
October	22063124	16,92	BQ.1.1.15	49 (0.2%)	Passed	Omicron5
October	22060884	16,89	BA.5.2	80 (0.3%)	Passed	Omicron5
October	22061044	16,81	BQ.1	22 (0.1%)	Passed	Omicron5
October	22060752	16,79	BQ.1.1	52 (0.2%)	Passed	Omicron5
October	22061507	16,77	BE.1	118 (0.4%)	Passed	Omicron5
October	22062060	16,7	BA.5.1	76 (0.3%)	Passed	Omicron5
October	22062600	16,65	BF.7	18 (0.1%)	Passed	Omicron5
October	22061468	16,59	BQ.1.15	78 (0.3%)	Passed	Omicron5
October	22060776	16,41	BF.14	183 (0.6%)	Passed	Omicron5
October	22061190	16,31	BQ.1.1	41 (0.1%)	Passed	Omicron5
October	22064173	16,29	BA.5.1	77 (0.3%)	Passed	Omicron5
October	22063128	16,28	BQ.1.2	84 (0.3%)	Passed	Omicron5
October	22061046	16,24	BA.5.1	345 (1.2%)	Passed	Omicron5
October	22061502	16,23	BL.2	424 (1.4%)	Passed	Omicron2
October	22064443	16,19	BM.1.1.3	692 (2.3%)	Passed	Omicron2
October	22064229	16,16	BA.5.2.1	2428 (8.1%)	Failed	Omicron5
October	22061087	16,12	BQ.1.10	551 (1.8%)	Passed	Omicron5
October	22061414	16,07	BA.5.2	77 (0.3%)	Passed	Omicron5
October	22060946	16,02	XBB.1.4	118 (0.4%)	Passed	Ricombinante
October	22061410	15,52	BF.5	25 (0.1%)	Passed	Omicron5
October	22060887	15,46	BE.1	118 (0.4%)	Passed	Omicron5
October	22061860	15,24	BA.5.2	24 (0.1%)	Passed	Omicron5
October	22061340	15,23	BQ.1.1.3	26 (0.1%)	Passed	Omicron5
October	22062897	14,92	BA.5.1	119 (0.4%)	Passed	Omicron5
October	22064220	14,9	BF.7.26	37 (0.1%)	Passed	Omicron5
October	22064439	14,78	BA.5.2.16	101 (0.3%)	Passed	Omicron5
October	22062788	14,73	XBB.1.4	22 (0.1%)	Passed	Ricombinante
October	22062419	14,69	BA.5.1.5	79 (0.3%)	Passed	Omicron5
October	22060945	14,67	XBB.1.4	63 (0.2%)	Passed	Ricombinante
October	22060881	14,59	BF.5	65 (0.2%)	Passed	Omicron5
October	22061859	14,49	BA.5.2	59 (0.2%)	Passed	Omicron5
October	22063822	14,48	BQ.1.1	69 (0.2%)	Passed	Omicron5
October	22061105	14,37	BA.4.6	64 (0.2%)	Passed	Omicron4
October	22061597	14,24	BA.5.1	88 (0.3%)	Passed	Omicron5
October	22063324	14,21	BA.5.2	75 (0.3%)	Passed	Omicron5
October	22061166	14,13	BA.5.1	53 (0.2%)	Passed	Omicron5
October	22061409	14,12	BA.5.1	16 (0.1%)	Passed	Omicron5
October	22061349	14,05	BA.5.2.1	19 (0.1%)	Passed	Omicron5
October	22063819	14,05	BA.5.1	63 (0.2%)	Passed	Omicron5
October	22064218	13,88	BA.5.1	1795 (6.0%)	Failed	Omicron5
October	22060947	13,77	BQ.1.1	52 (0.2%)	Passed	Omicron5
October	22061158	13,47	BQ.1.10	68 (0.2%)	Passed	Omicron5
October	22064183	13,39	BA.5.2	68 (0.2%)	Passed	Omicron5
October	22062792	13,38	BA.5.2	39 (0.1%)	Passed	Omicron5
October	22062049	13,23	BA.5.1	54 (0.2%)	Passed	Omicron5
October	22063127	13,06	BF.7.6	72 (0.2%)	Passed	Omicron5
October	22063820	12,8	BA.5.1	73 (0.2%)	Passed	Omicron5
October	22062791	12,56	BA.5.2	82 (0.3%)	Passed	Omicron5
October	22061503	12,51	BQ.1.2	70 (0.2%)	Passed	Omicron5
October	22061598	12,39	BA.5.1.10	24 (0.1%)	Passed	Omicron5
October	22061052	12,35	BF.10	339 (1.1%)	Passed	Omicron5
October	22061050	11,68	BA.4.6	70 (0.2%)	Passed	Omicron4
October	22063129	11,67	BQ.1.1	55 (0.2%)	Passed	Omicron5
October	22061157	11,45	BQ.1.10	91 (0.3%)	Passed	Omicron5

Supplementary Table S6

Month	Sample	Valore CT	Lineage	N_consensus	QC_status	Variante
November	22065829	30,91	BA.5	2666 (9.0%)	Failed	Omicron5
November	22067583	28,73	BA.2.10.1	1119 (3.8%)	Passed	Omicron2
November	22065438	27,69	BA.5.2	4428 (14.9%)	Failed	Omicron5
November	22069518	27,66	BA.5.1.10	1430 (4.8%)	Passed	Omicron5
November	22064777	27,64	BA.5.9	1219 (4.1%)	Passed	Omicron5
November	22069513	27,42	XBB	723 (2.4%)	Passed	Omicron2
November	22067585	27,27	BF.7.6	723 (2.4%)	Passed	Omicron5
November	22065704	27,2	BA.5.1.10	384 (1.3%)	Passed	Omicron5
November	22068109	26,96	XBB	709 (2.4%)	Passed	Omicron2
November	22068111	26,85	XBB	583 (2.0%)	Passed	Omicron2
November	22064772	26,7	BQ.1	817 (2.7%)	Passed	Omicron5
November	22065717	26,59	BN.1.3	855 (2.9%)	Passed	Omicron2
November	22065706	26,27	BQ.1	726 (2.4%)	Passed	Omicron5
November	22065739	26,23	BA.5.1	666 (2.2%)	Passed	Omicron5
November	22065701	26,17	BA.5.1.10	633 (2.1%)	Passed	Omicron5
November	22067586	25,99	BQ.1	419 (1.4%)	Passed	Omicron5
November	22069060	25,87	BQ.1.1	531 (1.8%)	Passed	Omicron5
November	22068091	25,68	BA.5.2.1	630 (2.1%)	Passed	Omicron5
November	22069045	25,28	XBB.1.4	870 (2.9%)	Passed	Omicron2
November	22065977	25,22	BQ.1.1	1739 (5.8%)	Failed	Omicron5
November	22064944	25,18	BQ.1.1.11	1729 (5.8%)	Failed	Omicron5
November	22065727	25,11	BA.5.9	545 (1.8%)	Passed	Omicron5
November	22065713	25,07	BQ.1	480 (1.6%)	Passed	Omicron5
November	22065720	24,94	BA.5.9	601 (2.0%)	Passed	Omicron5
November	22067263	24,82	XBB.1.4	546 (1.8%)	Passed	Ricombinante
November	22069512	24,76	XBB.1.4	607 (2.0%)	Passed	Ricombinante
November	22065707	24,62	BF.7	556 (1.9%)	Passed	Omicron5
November	22068161	24,5	BA.5.2	296 (1.0%)	Passed	Omicron5
November	22065496	24,4	BQ.1.1	1371 (4.6%)	Passed	Omicron5
November	22065818	24,32	XBB	672 (2.3%)	Passed	Omicron2
November	22069521	24,22	BF.7	422 (1.4%)	Passed	Omicron5
November	22065733	24,13	DB.2	230 (0.8%)	Passed	Omicron5
November	22067264	24,02	BA.5.1	2112 (7.1%)	Failed	Omicron5
November	22068482	23,54	XBF	2803 (9.4%)	Failed	Ricombinante
November	22065740	23,4	XBB.1.4	296 (1.0%)	Passed	Ricombinante
November	22064773	23,37	BQ.1.1.47	304 (1.0%)	Passed	Omicron5
November	22068479	22,91	BQ.1.1	638 (2.1%)	Passed	Omicron5
November	22069051	22,67	BF.7	870 (2.9%)	Passed	Omicron5
November	22065827	22,65	BQ.1	324 (1.1%)	Passed	Omicron5
November	22069517	22,47	BQ.1.1	238 (0.8%)	Passed	Omicron5
November	22069514	22,42	BM.1.1.3	358 (1.2%)	Passed	Omicron2
November	22068489	22,3	BQ.1.1.3	817 (2.7%)	Passed	Omicron5
November	22065760	22,28	BQ.1.1.23	570 (1.9%)	Passed	Omicron5
November	22067261	22,25	BQ.1.1.18	279 (0.9%)	Passed	Omicron5
November	22069520	22,25	BQ.1.1	271 (0.9%)	Passed	Omicron5
November	22069043	22,08	BF.27	587 (2.0%)	Passed	Omicron5
November	22064774	21,92	BQ.1	242 (0.8%)	Passed	Omicron5
November	22067460	21,76	CK.2.1	183 (0.6%)	Passed	Omicron5
November	22064986	21,32				
November	22067262	21,2	XBB.1	195 (0.7%)	Passed	Ricombinante
November	22065725	21,06	XBB.1.4	74 (0.2%)	Passed	Ricombinante
November	22065814	20,97	BA.5.1	1007 (3.4%)	Passed	Omicron5
November	22065750	20,92	BQ.1.1.18	173 (0.6%)	Passed	Omicron5
November	22067587	20,8	BQ.1.1.18	118 (0.4%)	Passed	Omicron5
November	22069054	20,61	BQ.1.1	875 (2.9%)	Passed	Omicron5
November	22065440	20,57	BF.3	1026 (3.4%)	Passed	Omicron5
November	22069052	20,56	BF.7	678 (2.3%)	Passed	Omicron5
November	22065721	20,35	XBB.1	308 (1.0%)	Passed	Ricombinante
November	22065761	20,26	BQ.1.1.23	158 (0.5%)	Passed	Omicron5
November	22066027	20,25	BQ.1.1	1026 (3.4%)	Passed	Omicron5
November	22067457	20,2	BA.5.2.1	275 (0.9%)	Passed	Omicron5
November	22067588	20,19	BQ.1.1.18	155 (0.5%)	Passed	Omicron5
November	22064947	20,11				
November	22068088	20,06	XBB.1.4	122 (0.4%)	Passed	Ricombinante
November	22067584	20	BQ.1.1.18	17 (0.1%)	Passed	Omicron5
November	22065813	19,73	BA.5.2.1	622 (2.1%)	Passed	Omicron5
November	22065743	19,68	BE.4.1	220 (0.7%)	Passed	Omicron5
November	22069074	19,6	BN.1.3.1	2093 (7.0%)	Failed	Omicron2
November	22069044	19,54	BF.7	682 (2.3%)	Passed	Omicron5
November	22064769	19,51	XBB.1.4	291 (1.0%)	Passed	Ricombinante
November	22065988	19,49	BF.14	693 (2.3%)	Passed	Omicron5
November	22065746	19,45	XBB.1.4	172 (0.6%)	Passed	Ricombinante
November	22065731	19,3	DB.2	120 (0.4%)	Passed	Omicron5
November	22067458	19,25	CL.1.3	318 (1.1%)	Passed	Omicron5
November	22068100	19,21	BQ.1.1	89 (0.3%)	Passed	Omicron5
November	22065756	19,08	BQ.1.1.23	308 (1.0%)	Passed	Omicron5
November	22065451	19,05	BF.7	610 (2.0%)	Passed	Omicron5
November	22065982	19,04	CQ.1	584 (2.0%)	Passed	Omicron5
November	22065744	19,02	BQ.1.1	612 (2.1%)	Passed	Omicron5
November	22064771	18,66	Unassigned	12321 (41.3%)	Failed	Omicron
November	22066025	18,57	BA.5.2	215 (0.7%)	Passed	Omicron5
November	22065702	18,39	XBB.1.4	117 (0.4%)	Passed	Ricombinante
November	22065755	18,3	BQ.1.1	162 (0.5%)	Passed	Omicron5
November	22065711	18,1	BF.5	64 (0.2%)	Passed	Omicron5
November	22065759	18,1	BQ.1.1.29	31 (0.1%)	Passed	Omicron5
November	22065735	18,09	BQ.1.1.3	49 (0.2%)	Passed	Omicron5
November	22065439	18,08	BN.1.3.1	2833 (9.5%)	Failed	Omicron2
November	22065506	18,08	BQ.1.1	705 (2.4%)	Passed	Omicron5
November	22065728	18,04	BA.5.9	118 (0.4%)	Passed	Omicron5
November	22067252	18,01	BA.5.1.10	119 (0.4%)	Passed	Omicron5
November	22064795	17,98	XBB.1.4	220 (0.7%)	Passed	Ricombinante
November	22064951	17,93				
November	22069519	17,88	BQ.1.1	154 (0.5%)	Passed	Omicron5
November	22067466	17,77	BQ.1.13	13 (0.0%)	Passed	Omicron5
November	22065708	17,76	BF.7	394 (1.3%)	Passed	Omicron5
November	22068095	17,75	BQ.1.1	108 (0.4%)	Passed	Omicron5
November	22065741	17,71	BE.4.2	150 (0.5%)	Passed	Omicron5
November	22064770	17,67	BA.5.1	316 (1.1%)	Passed	Omicron5
November	22065714	17,67	BA.5.1	18 (0.1%)	Passed	Omicron5
November	22067461	17,49	CL.1.3	69 (0.2%)	Passed	Omicron5
November	22067255	17,45	BN.1.3.1	279 (0.9%)	Passed	Omicron2
November	22067590	17,39	BF.7.4.2	68 (0.2%)	Passed	Omicron5
November	22065736	17,24	BQ.1.1	69 (0.2%)	Passed	Omicron5
November	22067251	17,24	BA.5.1.10	63 (0.2%)	Passed	Omicron5
November	22069073	17,18	BQ.1.1.6	576 (1.9%)	Passed	Omicron5

November	22065703	17,05	BF.5	116 (0.4%)	Passed	Omicron5
November	22065724	17,03	BQ.1	16 (0.1%)	Passed	Omicron5
November	22069515	16,67	BM.1.1.3	400 (1.3%)	Passed	Omicron2
November	22065705	16,62	BF.7	191 (0.6%)	Passed	Omicron5
November	22065738	16,54	BN.1.3	314 (1.1%)	Passed	Omicron2
November	22065710	16,49	EQ.1	17 (0.1%)	Passed	Omicron5
November	22065734	16,49	XBB.1.4	76 (0.3%)	Passed	Ricombinante
November	22068480	16,48	BF.7.1	555 (1.9%)	Passed	Omicron5
November	22065729	16,2	BA.5.1	485 (1.6%)	Passed	Omicron5
November	22064941	16,14	XBB.1.4	733 (2.5%)	Passed	Ricombinante
November	22065445	15,94	BA.5.1.10	717 (2.4%)	Passed	Omicron5
November	22068486	15,88	XBF	1033 (3.5%)	Passed	Ricombinante
November	22064945	15,82				
November	22065715	15,78	BQ.1.1	15 (0.1%)	Passed	Omicron5
November	22068089	15,74	BQ.1.1	36 (0.1%)	Passed	Omicron5
November	22065723	15,72	BQ.1.1	22 (0.1%)	Passed	Omicron5
November	22067582	15,41	XBB.1.4	69 (0.2%)	Passed	Ricombinante
November	22067250	15,25	BA.5.2	67 (0.2%)	Passed	Omicron5
November	22064942	15,13				
November	22065712	15,11	BM.1.1.3	267 (0.9%)	Passed	Omicron2
November	22065719	15,01	BA.5.9	27 (0.1%)	Passed	Omicron5
November	22065752	14,91	BF.3	19 (0.1%)	Passed	Omicron5
November	22065749	14,83	XBB.1.4	20 (0.1%)	Passed	Ricombinante
November	22065732	14,77	XBB.1.4	68 (0.2%)	Passed	Ricombinante
November	22064775	14,68	BA.5.2	65 (0.2%)	Passed	Omicron5
November	22065737	14,64	BF.5	68 (0.2%)	Passed	Omicron5
November	22065709	14,62	BF.10	74 (0.2%)	Passed	Omicron5
November	22064956	14,6				
November	22069053	14,52	XBB.1.4	156 (0.5%)	Passed	Ricombinante
November	22064768	14,5	BN.1.3	484 (1.6%)	Passed	Omicron2
November	22068492	14,33	BF.7	93 (0.3%)	Passed	Omicron5
November	22065833	14,32	BF.11	71 (0.2%)	Passed	Omicron5
November	22065745	14,19	XBB.1.4	12 (0.0%)	Passed	Ricombinante
November	22065753	14,16	XBB.1.4	112 (0.4%)	Passed	Ricombinante
November	22065441	13,99	BF.7	412 (1.4%)	Passed	Omicron5
November	22068488	13,88	BA.5.2.18	68 (0.2%)	Passed	Omicron5
November	22065810	13,81	BF.7.4.2	118 (0.4%)	Passed	Omicron5
November	22065444	13,58	BA.5.1.10	610 (2.0%)	Passed	Omicron5
November	22065747	13,57	BQ.1.1	14 (0.0%)	Passed	Omicron5
November	22065751	13,44	BF.7	68 (0.2%)	Passed	Omicron5
November	22064943	13,41				
November	22068481	13,06	BN.1.3	652 (2.2%)	Passed	Omicron2
November	22068487	13,02	BQ.1.15	286 (1.0%)	Passed	Omicron5
November	22065742	12,82	BF.7.9	21 (0.1%)	Passed	Omicron5
November	22065718	12,72	BQ.1.1	69 (0.2%)	Passed	Omicron5
November	22065722	12,67	BQ.1.1	35 (0.1%)	Passed	Omicron5
November	22065757	12,65	BM.1.1	276 (0.9%)	Passed	Omicron2
November	22067254	12,63	BQ.1.13	38 (0.1%)	Passed	Omicron5
November	22065816	12,48	BQ.1.1.18	76 (0.3%)	Passed	Omicron5
November	22067259	12,48	XBB.1.4	68 (0.2%)	Passed	Ricombinante
November	22065843	12,33	BF.5	41 (0.1%)	Passed	Omicron5
November	22065758	12,3	XBB.1.4	78 (0.3%)	Passed	Ricombinante
November	22065716	11,53	BQ.1.10	68 (0.2%)	Passed	Omicron5
November	22065754	11,2	XBB.1.4	69 (0.2%)	Passed	Ricombinante
November	22065726	10,97	BQ.1.2	68 (0.2%)	Passed	Omicron5
November	22068491	10,48	BF.7	129 (0.4%)	Passed	Omicron5
November	22065437	10,27	BQ.1.1	14 (0.0%)	Passed	Omicron5

Supplementary Table S7

Month	Sample	Valore CT	Lineage	N_consensus	QC_status	Variante
December	22071669	28,62	BA.2	3913 (13.1%)	Failed	Omicron2
December	22070197	28,2	BA.5	7951 (26.6%)	Failed	Omicron5
December	22071670	28,1	XBB.1.4	1223 (4.1%)	Passed	Ricombinante
December	22071660	28	BE.1.1	1740 (5.8%)	Failed	Omicron5
December	22071627	27,96	BQ.1	1544 (5.2%)	Failed	Omicron5
December	22072524	27,48	BQ.1	1570 (5.3%)	Failed	Omicron5
December	22070787	27,36	BQ.1	2107 (7.1%)	Failed	Omicron5
December	22071651	27,13	BF.7	1203 (4.0%)	Passed	Omicron5
December	22071630	27,06	BQ.1.1	1240 (4.2%)	Passed	Omicron5
December	22071629	26,84	BQ.1	1435 (4.8%)	Passed	Omicron5
December	22072839	26,66	BA.5.2.1	760 (2.6%)	Passed	Omicron5
December	22071668	26,62	XBB.1.4	881 (3.0%)	Passed	Omicron2
December	22071658	26,55	BA.5.2.47	781 (2.6%)	Passed	Omicron5
December	22072832	26,31	BA.2	905 (3.0%)	Passed	Ricombinante
December	22070801	26,13	BQ.1	3270 (11.0%)	Failed	Omicron5
December	22071662	25,8	BA.5	5406 (18.1%)	Failed	Omicron5
December	22070191	25,79	XBF	1148 (3.9%)	Passed	Ricombinante
December	22071672	25,51	BQ.1	1055 (3.5%)	Passed	Omicron5
December	22071654	25,32	BQ.1.13.1	1158 (3.9%)	Passed	Omicron5
December	22071641	25,21	BF.7.1	761 (2.6%)	Passed	Omicron5
December	22071677	24,87	BQ.1	870 (2.9%)	Passed	Omicron5
December	22072526	24,78	BQ.1	1095 (3.7%)	Passed	Omicron5
December	22071656	24,53	XBB.1.4	2244 (7.5%)	Failed	Ricombinante
December	22071671	24,52	BQ.1.1	587 (2.0%)	Passed	Omicron5
December	22070687	24,21	BN.1.3.1	1668 (5.6%)	Failed	Omicron2
December	22070791	23,93	BF.7	1161 (3.9%)	Passed	Omicron5
December	22070783	23,6	BQ.1.1	1070 (3.6%)	Passed	Omicron5
December	22072527	23,19	BQ.1	1383 (4.6%)	Passed	Omicron5
December	22071644	22,74	BQ.1	660 (2.2%)	Passed	Omicron5
December	22071621	22,47	BF.7	647 (2.2%)	Passed	Omicron5
December	22071614	22,14	BQ.1.1	441 (1.5%)	Passed	Omicron5
December	22072529	22,14	BQ.1.1	856 (2.9%)	Passed	Omicron5
December	22069647	21,58	BQ.1.1	409 (1.4%)	Passed	Omicron5
December	22072528	21,4	CK.2.1.1	508 (1.7%)	Passed	Omicron5
December	22071665	21,36	XBF	1278 (4.3%)	Passed	Ricombinante
December	22070685	21,26	BQ.1.5	200 (0.7%)	Passed	Omicron5
December	22071158	21,06	BR.3	1076 (3.6%)	Passed	Omicron2
December	22071675	21,03	BQ.1.1.18	400 (1.3%)	Passed	Omicron5
December	22071634	20,98				
December	22070772	20,73	BF.7	656 (2.2%)	Passed	Omicron5
December	22071618	20,48	BQ.1.1	347 (1.2%)	Passed	Omicron5
December	22071650	20,36	BQ.1	254 (0.9%)	Passed	Omicron5
December	22070684	20,11	BQ.1.1	373 (1.3%)	Passed	Omicron5
December	22071666	19,93	BQ.1.1	635 (2.1%)	Passed	Omicron5
December	22072836	19,8	XBB.1.4	558 (1.9%)	Passed	Ricombinante
December	22070779	19,73	BF.7	357 (1.2%)	Passed	Omicron5
December	22070188	19,65	XBB.1.4	481 (1.6%)	Passed	Ricombinante
December	22072525	19,62	BQ.1	673 (2.3%)	Passed	Omicron5
December	22071632	19,6				
December	22071619	19,59	BQ.1.1	509 (1.7%)	Passed	Omicron5
December	22072559	19,38	BQ.1.8	849 (2.8%)	Passed	Omicron5
December	22071625	19,26	XBF	917 (3.1%)	Passed	Ricombinante
December	22071626	19,2	BF.7	860 (2.9%)	Passed	Omicron5
December	22072835	18,98	BQ.1.10.1	207 (0.7%)	Passed	Omicron5
December	22070771	18,92	BF.7	489 (1.6%)	Passed	Omicron5
December	22071649	18,85	BQ.1.1.31	583 (2.0%)	Passed	Omicron5
December	22070682	18,47	BQ.1.1.18	280 (0.9%)	Passed	Omicron5
December	22071642	18,42	BF.7.1	241 (0.8%)	Passed	Omicron5
December	22070187	18,35	BF.7	627 (2.1%)	Passed	Omicron5
December	22071647	18,34	BF.7	701 (2.3%)	Passed	Omicron5
December	22071645	18,18	BQ.1.8	259 (0.9%)	Passed	Omicron5
December	22070186	18,17	BQ.1.1	387 (1.3%)	Passed	Omicron5
December	22071622	18,13	XBF	536 (1.8%)	Passed	Ricombinante
December	22071636	18,13				
December	22071631	18,04				
December	22071653	17,72	BQ.1.13.1	592 (2.0%)	Passed	Omicron5
December	22070686	17,7	BQ.1.5	221 (0.7%)	Passed	Omicron5
December	22069645	17,63	BF.7	579 (1.9%)	Passed	Omicron5
December	22071664	17,39	BA.5.1.5	256 (0.9%)	Passed	Omicron5
December	22070774	17,29	BN.1.3.1	867 (2.9%)	Passed	Omicron2
December	22071657	16,99	XBF	604 (2.0%)	Passed	Ricombinante
December	22070683	16,95	BQ.1.1	525 (1.8%)	Passed	Omicron5
December	22071611	16,88	BQ.1.1	170 (0.6%)	Passed	Omicron5
December	22071620	16,66	BA.5.1	3157 (10.8%)	Failed	Omicron5

December	22069644	16,37	BQ.1.1.4	72 (0.2%)	Passed	Omicron5
December	22072523	16,35	BQ.1.1	385 (1.3%)	Passed	Omicron5
December	22071613	16,24	BF.7	306 (1.0%)	Passed	Omicron5
December	22072837	16,21	BN.1.2	360 (1.2%)	Passed	Omicron2
December	22070190	16,13	XBB.1	69 (0.2%)	Passed	Ricombinante
December	22071615	16,04	XBB.1.4	355 (1.2%)	Passed	Ricombinante
December	22070688	15,97	BN.1.3.1	660 (2.2%)	Passed	Omicron2
December	22070441	15,62	BQ.1.1.18	74 (0.2%)	Passed	Omicron5
December	22071624	15,6	BQ.1.1	216 (0.7%)	Passed	Omicron5
December	22071635	15,56				
December	22070785	15,49	BN.1.3	399 (1.3%)	Passed	Omicron2
December	22071639	15,49				
December	22071643	15,19	BQ.1.8	345 (1.2%)	Passed	Omicron5
December	22071640	15,03	BF.7.1	310 (1.0%)	Passed	Omicron5
December	22070446	14,86	BQ.1.1	202 (0.7%)	Passed	Omicron5
December	22072522	14,85	BA.5.2.63	97 (0.3%)	Passed	Omicron5
December	22071676	14,64	BA.5.1	74 (0.2%)	Passed	Omicron5
December	22070201	14,46	BN.1.3.1	359 (1.2%)	Passed	Omicron2
December	22072532	13,99	BR.3	343 (1.1%)	Passed	Omicron2
December	22071648	13,96	BQ.1.1.53	68 (0.2%)	Passed	Omicron5
December	22071617	13,67	BN.1.3.1	359 (1.2%)	Passed	Omicron2
December	22070194	13,54	BQ.1.1	68 (0.2%)	Passed	Omicron5
December	22070192	13,29	BF.7.4.2	74 (0.2%)	Passed	Omicron5
December	22071637	12,58				
December	22072833	12,09	XBF	343 (1.1%)	Passed	Ricombinante
December	22069640	11,34	BF.7.12	104 (0.3%)	Passed	Omicron5
December	22071659	11,28	BQ.1.1.47	69 (0.2%)	Passed	Omicron5
December	22071638	11,2				
December	22069641	10,45	BQ.1.1	70 (0.2%)	Passed	Omicron5
